# Revisiting the potential of upconversion nanoparticles in biomedical applications: Advances and emerging perspectives

**DOI:** 10.1016/j.mtbio.2026.103352

**Published:** 2026-06-11

**Authors:** Faezeh Ghorbanizamani, Hichem Moulahoum, David John Dmonte, Kalim Deshmukh

**Affiliations:** a Department of Biochemistry, Faculty of Science, Ege University, Bornova, Izmir, 35100, Türkiye; b New Technologies Research Centre, University of West Bohemia in Pilsen, Univerzitní 2732/8, Pilsen, 30100, Czech Republic

**Keywords:** Upconversion nanoparticles, Biomedicine, Luminescence, Bioimaging, Biosensing, Theranostics, Drug delivery, Optogenetics

## Abstract

Upconversion nanoparticles (UCNPs) have evolved from niche photophysical systems into multifunctional platforms with growing relevance in biomedicine. Their ability to convert near-infrared (NIR) excitation into higher-energy emission enables deep tissue interrogation with minimal background and high photostability, offering clear advantages over conventional probes. This review revisits the field from a design-oriented perspective, reflecting how recent advances in synthesis, photophysical understanding, and nanoengineering have reshaped the relationship between UCNP structure and biomedical performance. Combining properties and applications highlight how rational control over architecture and interfaces governs functionality in complex biological environments. This evolving framework is discussed across major application domains, alongside emerging directions that extend UCNPs beyond passive imaging toward active and programmable systems. Despite substantial progress, key challenges persist in achieving high brightness under safe excitation, ensuring predictable biological behavior, enabling safe clearance, and improving reproducibility. Addressing these limitations through integrated material and system-level design will be critical for translating UCNPs into reliable biomedical technologies.

## Graphical abstract

**Figure:**
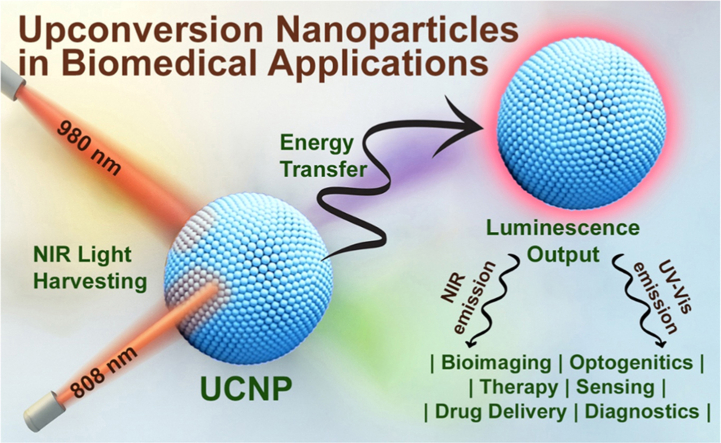

## 1 Introduction

Over the past three decades, nanotechnology has profoundly shaped the biomedical landscape by offering innovative platforms for disease diagnosis, therapy, and monitoring at previously inaccessible spatial and temporal resolutions. Nanoparticles, owing to their tunable size, morphology, and surface chemistry, have been engineered to perform tasks ranging from targeted drug delivery to high-resolution bioimaging. Among the most widely explored nanomaterials, metal and semiconductor nanoparticles, liposomes, micelles, and polymeric nanocarriers have found substantial use in preclinical and clinical research [1]. Despite their promise, however, these systems are often limited by instability under physiological conditions, poor photostability, broad emission spectra, limited tissue penetration, and in some cases, toxicity concerns that restrict their safe use in vivo.

Fluorescence imaging has been central to biomedical research, but conventional organic fluorophores suffer from rapid photobleaching and limited signal-to-noise ratios because of autofluorescence from biological materials. Semiconductor quantum dots partially overcame these challenges with higher photostability and size-tunable emission, yet concerns over heavy-metal toxicity and blinking effects have restricted their biomedical translation [2]. These limitations spurred the search for alternative luminescent nanomaterials that combine brightness, photostability, and biocompatibility with minimal background interference.

A transformative breakthrough came with the development of upconversion nanoparticles (UCNPs), a class of luminescent materials able of transforming near-infrared (NIR) excitation waves into visible or ultraviolet (UV) emission through multiphoton processes mediated by lanthanide ions embedded in crystalline host lattices. The anti-Stokes emission of UCNPs is unique in that it enables excitation with low-energy NIR photons, wavelengths that penetrate deeper into biological tissues while simultaneously avoiding autofluorescence and minimizing photodamage [3,4]. This property, together with the long luminescence lifetimes and sharp emission bands associated with lanthanide f-f transitions, distinguished UCNPs from conventional fluorophores and opened new avenues for sensitive biomedical detection and imaging.

Historically, the concept of photon upconversion was first recognized in bulk lanthanide-doped crystals during the 1950s and 1960s [5]. Researchers observed that certain lanthanide ions, such as Erbium (Er^3+^) and Thulium (Tm^3+^), could absorb multiple photons sequentially and produce light of higher energy. For several decades, upconversion remained primarily a topic of solid-state physics and laser science, with applications in infrared detectors, solid-state lasers, and optical communication [6,7]. It was not until the 1990s and early 2000s that the miniaturization of these materials into nanoscale colloids (True UCNPs) enabled their exploration in chemistry and biology [8]. The pioneering synthesis of β-NaYF_4_ nanocrystals doped with Ytterbium (Yb^3+^) and Er^3+^ represented a major milestone, as NaYF_4_ remains one of the most competent host lattices for photon upconversion [9]. This development laid the foundation for biomedical applications by combining strong upconversion emission with the possibility of colloidal dispersion after suitable surface engineering.

From this point forward, UCNPs rapidly evolved into a central research focus in nanobiotechnology. Their NIR excitation wavelengths (typically 808 or 980 nm) enable deeper tissue penetration than visible excitation sources [10], while biological autofluorescence is largely suppressed, resulting in improved signal-to-noise ratios in imaging and sensing applications [11]. These advantages place UCNPs within a broader class of NIR-responsive nanomaterials that have been developed to overcome the limitations of conventional optical probes. In addition to UCNPs, systems such as NIR-II organic fluorophores, aggregation-induced emission luminogens (AIEgens), semiconductor quantum dots, and carbon-based nanomaterials have been explored for biomedical applications, each leveraging NIR excitation or emission to achieve deeper tissue imaging and improved contrast [12,13]. However, these materials differ significantly in their photophysical properties, stability, and functional capabilities.

In the case of UCNPs, their exceptional photostability enables prolonged imaging and tracking experiments [14], while their narrow emission peaks permit multiplexed detection with minimal spectral overlap [15,16]. Furthermore, their microsecond-to-millisecond luminescence lifetimes enable time-gated detection strategies that effectively suppress short-lived background fluorescence [17,18]. Beyond optical performance, UCNPs can be engineered for multifunctionality. By integrating magnetic dopants or hybridizing with superparamagnetic nanoparticles, UCNP-based probes can offer combined optical and magnetic resonance imaging (MRI) capabilities [16,[19], [20], [21], [22], [23]], while surface modification through silica shells, polymer coatings, or ligand exchange enables water dispersibility, biocompatibility, and specific molecular recognition [21,24].

Despite the rapid development of these NIR-responsive systems, their performance is highly application-dependent. For example, organic NIR fluorophores and AIEgens often provide higher brightness and simpler implementation for imaging, whereas UCNPs offer distinct advantages in applications requiring long-lived emission, low background interference, and remote activation through anti-Stokes processes [25]. Similarly, carbon-based nanomaterials and quantum dots provide scalable and tunable platforms but may suffer from broad emission profiles or toxicity-related concerns. A comparative understanding of these material classes is therefore essential to position UCNPs within the broader landscape of NIR nanomaterials and to identify scenarios where their unique properties provide clear functional advantages (Table 1).

**Table 1:** Comparative overview of UCNPs and other NIR-responsive nanomaterials.

Material System	Excitation /Emission Range	Key Advantages	Key Limitations	Typical Applications
**Upconversion nanoparticles (UCNPs)**	NIR (typically 808-980 nm) → Visible/UV	Anti-Stokes emission; low autofluorescence; long lifetimes; high photostability; multiplexing capability [26]	Low absorption cross-section and low quantum yield; limited brightness at low irradiance; need for improved efficiency [26]	Bioimaging, biosensing, theranostics, optogenetics [26]
**NIR-II fluorophores (Organic dyes)**	NIR-I/NIR-II (700-1700 nm) → NIR-II emission	Deep tissue penetration; reduced scattering and autofluorescence; high spatial resolution; fast imaging capability [27,28]	Photobleaching; short lifetimes; complex synthesis; limited clinical translation and stability issues [27,28]	In vivo imaging, vascular imaging, surgical guidance [28,29]
**Aggregation-induced emission luminogens (AIEgens)**	Visible/NIR excitation → Visible/NIR emission	High brightness in aggregated state; improved photostability; flexible molecular engineering [12,28]	Still developing; limited clinical translation; challenges in optimization and targeting [12,28]	Bioimaging, biosensing, phototherapy [12]
**Carbon-based nanomaterials (carbon dots, graphene derivatives)**	UV/Visible/NIR excitation → Visible/NIR emission	Low toxicity; good biocompatibility; tunable photoluminescence; chemical stability [26,29]	Broad emission spectra; limited multiplexing; environmental sensitivity; need for red-shifted emission for deeper imaging [26]	Bioimaging, sensing, drug delivery [26,27]
**Semiconductor quantum dots (QDs)**	UV/Visible excitation → Visible/NIR emission	High quantum yield; narrow emission bands; tunable size-dependent emission [26]	Toxicity concerns (Cd, Pb, Hg, etc.); instability; autofluorescence; limited clinical translation [26,29]	Bioimaging, multiplexed sensing, diagnostics [26,27]

The first demonstrations of UCNPs in biomedical applications appeared in the mid-2000s, when researchers employed them for deep-tissue luminescence imaging in small animal models [30]. Around the same period, UCNPs were introduced as labels in lateral flow immunoassays, offering higher sensitivity compared to gold nanoparticles or organic dyes [31]. Shortly thereafter, UCNP-based nanosystems were explored for photodynamic therapy (PDT), where NIR-excited UCNPs activated photosensitizers to generate cytotoxic singlet oxygen for localized tumor destruction [32,33]. These early studies established the core biomedical value of UCNPs in applications requiring low background, deep penetration, and multiplex detection.

In the last decade, the biomedical scope of UCNPs has expanded remarkably. Their use in cancer diagnostics and therapy has grown, with UCNPs serving as both imaging probes and therapeutic mediators in multimodal theranostic platforms [34,35]. In biosensing, UCNPs have enabled ultrasensitive detection of proteins, nucleic acids, and small molecules through luminescence resonance energy transfer (LRET) and related mechanisms [36]. In bioimaging, UCNPs have proven valuable in long-term cell tracking, whole-animal imaging, and targeted subcellular visualization [37,38]. More recently, UCNPs have entered emerging frontiers such as super-resolution imaging [30], optogenetics [39], and nanoscale thermometry [40]. The breadth of these applications reflects not only the adaptability of UCNP design but also the increasing integration of UCNPs into complex biomedical platforms.

Despite this progress, the translation of UCNPs into clinical practice remains limited. Several challenges persist, including relatively low upconversion quantum yields under biologically safe power densities, synthetic difficulties in achieving uniform and monodisperse nanocrystals, and incomplete understanding of long-term biodistribution, clearance, and toxicity in vivo [41]. In addition, the complex surface chemistry required for biocompatibility and functionalization can introduce variability and batch-to-batch inconsistency [41]. While several reviews have addressed specific aspects of UCNPs, such as synthesis, optical mechanisms, or selected biomedical applications [[42], [43], [44]], there remains a need for a more integrated and critical analysis that connects material design to functional performance across biomedical settings.

In this context, revisiting UCNPs is both timely and necessary. Recent studies have moved the field beyond the demonstration of isolated luminescent probes toward the development of more integrated and application-oriented nanoplatforms, where optical performance, structural architecture, and interfacial engineering are deliberately coordinated to achieve specific biomedical functions [11,34,35]. At the same time, growing attention has been directed toward persistent limitations such as brightness under safe excitation, surface quenching, biological-medium stability, biodistribution, and translational reproducibility [41,43]. Accordingly, the present review aims to provide a structured and updated perspective by explicitly linking synthesis strategies, upconversion mechanisms, physicochemical and biological determinants, and surface engineering approaches to the resulting biomedical performance of UCNPs. Rather than treating applications as isolated examples, this review seeks to clarify how specific design choices govern function in sensing, imaging, therapy, and emerging smart biomedical systems, while also highlighting the major barriers that continue to limit realistic clinical translation.

## 2 Synthesis methods of upconversion nanoparticles

The synthesis of UCNPs is a decisive factor governing their luminescence efficiency, particle size, morphology, surface chemistry, and ultimately their performance in biomedical applications. Since UCNPs are intended for highly sensitive diagnostic and therapeutic tasks, the ability to reproducibly prepare high-quality nanocrystals with tailored properties has remained at the center of research efforts. There are several strategies used to prepare UCNPs with each of these carrying advantages and limitations in terms of crystallinity, scalability, surface compatibility, and suitability for downstream biomedical functionalization [45].

### 2.1 High-temperature solid-state reactions

The earliest demonstrations of photon upconversion in lanthanide-doped materials relied on high-temperature solid-state reactions, typically conducted at temperatures exceeding 800-1100°C [[46], [47], [48], [49]]. In these methods, stoichiometric mixtures of lanthanide oxides or nitrates are intimately ground with fluoride precursors such as NaF or NH_4_F, and then subjected to prolonged calcination to yield crystalline upconversion phosphors [[47], [48], [49], [50]]. These reactions often incorporate flux agents (e.g., H_3_BO_3_) to enhance crystallinity and luminescence intensity [51]. The resulting products are bulk powders with strong upconversion luminescence (UCL), which served as foundational platforms for investigating the influence of host lattices, dopant concentrations, and phonon energy effects on optical efficiency. For instance, studies on β-NaYF_4_ and related hosts revealed how high dopant loadings can induce quenching, and how lattice symmetry affects emission intensity [52,53]. However, from a biomedical perspective, these materials are largely unsuitable due to their large particle size, polydispersity, and poor aqueous dispersibility [54]. The lack of control over morphology, phase purity, and surface chemistry limits their utility in biological environments. Consequently, while solid-state synthesis remains valuable for cost-effective bulk production and fundamental optical studies, it has been largely supplanted by colloidal methods for biomedical-grade UCNPs. Nonetheless, many key insights into energy transfer dynamics, dopant-host interactions, and thermal defect formation were derived from these early materials, paving the way for more refined synthetic strategies [45]. As such, while high-temperature solid-state reactions provide highly crystalline materials that are valuable for understanding fundamental upconversion mechanisms, their limited control over particle size and surface properties restricts their direct applicability in biomedical systems requiring nanoscale, dispersible, and surface-functionalizable UCNPs.

### 2.2 Thermal decomposition

It is one of the most widely employed techniques for synthesizing fluoride-based UCNPs, offering precise control over size, shape, and phase purity. In this method, lanthanide precursors such as trifluoroacetates, acetylacetonates, or chlorides are dissolved in high-boiling organic solvents like oleic acid (OA), oleylamine (OM), and octadecene (ODE), and heated to 280-320°C under an inert atmosphere [[55], [56], [57]]. OA and OM act as surface-capping ligands, preventing aggregation and regulating nanocrystal growth, while ODE serves as the reaction medium [57,58]. This process promotes the formation of highly crystalline, monodisperse nanoparticles, typically ranging from 10 to 100 nm, with tunable shapes such as spheres, hexagonal prisms, or rods, depending on the precursor chemistry and reaction conditions [59,60]. Importantly, thermal decomposition readily yields the hexagonal β-NaYF_4_ phase, which is recognized as the most competent host lattice for photon upconversion [55,58]. The method also allows for doping flexibility, enabling the synthesis of various fluoride crystals such as LnF_3_, NaLnF_4_, and Na_5_Ln_9_F_32_ with tailored optical properties [55]. However, the hydrophobic OA coating renders the as-synthesized UCNPs dispersible only in non-polar solvents. For biomedical applications, post-synthetic adjustments such as silica coating, ligand exchange, or polymer encapsulation are required to achieve water solubility and biocompatibility [60,61]. Additionally, the need for high-temperature equipment and inert gas protection complicates scalability and increases production costs [61]. Despite these limitations, thermal decomposition remains the gold standard for producing UCNPs with superior luminescence efficiency. Such nanoparticles have been widely employed in in vivo imaging, targeted drug delivery, and tumor-specific diagnostics, following appropriate surface functionalization [60,61]. Therefore, while thermal decomposition enables precise control over crystallinity and optical performance, its reliance on hydrophobic surface ligands necessitates additional surface engineering to ensure compatibility with aqueous biological environments.

### 2.3 Hydrothermal and solvothermal routes

Hydrothermal and solvothermal methods offer versatile alternatives for synthesizing water-dispersible UCNPs, particularly when biomedical compatibility is a priority. In hydrothermal synthesis, lanthanide nitrates or chlorides are mixed with fluoride sources such as NaF or NH_4_F in aqueous media, and the solution is sealed in a Teflon-lined autoclave. Heating at 150-240°C under autogenous pressure for several hours promotes nucleation and crystallization of UCNPs [62,63]. Key specifications such as pH, precursor ratios, reaction time, and the presence of chelating molecules like EDTA, citrate, or polyethyleneimine (PEI) strongly influence particle size, morphology, and phase outcome [64,65]. Compared to thermal decomposition, hydrothermal synthesis is less equipment-intensive, operates at lower temperatures, and is more scalable [63]. Crucially, when hydrophilic ligands such as citric acid, poly(acrylic acid) (PAA), or polyethylene glycol (PEG) are introduced during synthesis process, the produced UCNPs are directly dissolvable in water, eliminating the need for post-synthetic surface modification [64,65]. However, hydrothermal methods often yield broader size distributions and may favor the creation of the cubic α-NaYF_4_ phase, which optically inefficient compared to the hexagonal β-phase [65]. To address this, researchers have developed two-step synthesis protocols, where an initial cubic phase is converted to the hexagonal form via thermal treatment or phase-directing additives [65].

Solvothermal synthesis extends this approach by using organic solvents such as ethanol or ethylene glycol, allowing reactions to proceed under lower pressures and offering greater control over morphology [65]. For example, UCNPs synthesized with EDTA as a chelating agent exhibit rod-like morphologies, while those prepared with OA form hexagonal nanorods, due to differences in ligand acidity and chain length [65]. Biomedical applications of hydrothermally and solvothermally prepared UCNPs are diverse. Citrate capped NaYF_4_:Yb,Er NPs have been utilized as intracellular nanothermometers, leveraging ratiometric luminescence to map temperature gradients in living cells [65]. Similarly, PEI- or PAA-coated UCNPs have served as multifunctional drug carriers, enabling real-time luminescence tracking of drug release and bioconjugation with targeting molecules [64]. These examples underscore how the hydrophilic nature, biocompatibility, and synthetic flexibility of hydrothermal and solvothermal methods make them ideal for direct integration into biological systems. Accordingly, the inherent hydrophilicity and synthetic versatility of hydrothermal and solvothermal approaches make them particularly advantageous for biomedical applications requiring direct aqueous dispersibility and simplified surface functionalization.

### 2.4 Co-precipitation

Among the simplest and most cost-effective synthesis routes for UCNPs is co-precipitation, in which lanthanide salts (e.g., nitrates or chlorides) are mixed with fluoride sources such as NaF or NH_4_F in aqueous or polar solvents at moderate temperatures [66]. The simultaneous precipitation of lanthanide fluorides leads to nanoparticle formation. This method can be conducted under relatively mild conditions (typically below 100°C) without specialized equipment, making it highly accessible and scalable [67,68]. The major challenge in co-precipitation lies in controlling crystallinity and phase purity. Products often comprise a mixture of cubic (α) and hexagonal (β) NaYF_4_ phases, and the initial crystallinity may be insufficient to yield strong UCL [67]. Post-synthetic annealing at elevated temperatures (400-600°C) can improve crystallinity and promote phase transformation to the β-phase, but it also risks particle aggregation and size growth [67].

To mitigate these issues, chelating agents and stabilizers such as citric acid, PEG, and polyvinylpyrrolidone (PVP) are often employed during synthesis to regulate nucleation, growth, and surface passivation, resulting in better control over particle dispersion and morphology [67]. Despite their lower luminescence efficiency compared to thermally decomposed UCNPs, co-precipitated nanoparticles have found successful biomedical applications. Their small size (often 10-30 nm) and hydrophilic surfaces make them exceptionally suitable for biosensing, drug delivery, and targeted imaging. For instance, citrate-capped NaYF_4_:Yb,Er UCNPs coupled with folate have been employed to selectively image cancer cells overexpressing folate receptors [67]. Similarly, PEG-modified UCNPs synthesized via co-precipitation have been applied in nucleic acid detection assays, where their low background signal enabled sensitive detection of DNA hybridization [69,70]. Co-precipitation remains a practical and biocompatible route for producing UCNPs tailored to biological environments, especially when surface hydrophilicity, low toxicity, and scalability are prioritized over peak luminescence performance. Thus, co-precipitation offers a favorable balance between scalability, hydrophilicity, and biocompatibility, although its comparatively lower crystallinity and luminescence efficiency must be considered when high optical performance is required.

### 2.5 Sol-gel processes

This approach is a flexible method for UCNPs synthesis, particularly when embedding them within silica or oxide matrices [66]. It implicates the hydrolysis and polycondensation of metal alkoxides or inorganic salts in solution, forming a homogeneous gel that is subsequently calcined at high temperatures to induce crystallization [71]. This method is especially advantageous for fabricating hybrid nanocomposites, thin films, and glass ceramics, as UCNPs can be incorporated into silica networks that offer mechanical stability, chemical protection, and surface functionalization potential [71,72]. However, obtaining colloidal UCNPs via sol-gel synthesis remains challenging. The high-temperature calcination required (typically 600-1000°C) can lead to particle growth, agglomeration, and loss of surface control, which in turn compromises luminescence efficiency compared to UCNPs prepared by thermal decomposition [72]. Additionally, the dense oxide matrix may partially quench upconversion emission due to phonon interactions, unless carefully engineered. Despite these limitations, sol-gel-derived UCNP composites have found promising biomedical applications. For instance, UCNP-silica hybrid systems have been developed as drug delivery platforms, where the silica matrix provides controlled release properties, while the UCNPs enable optical tracking of release events. Similarly, sol-gel films embedding UCNPs have been investigated as biosensing interfaces, combining luminescence detection with surface robustness and chemical inertness, making them suitable for implantable or wearable sensor platforms [72]. While sol-gel synthesis is less suited for producing monodisperse colloidal UCNPs, it remains a valuable route for structural integration, optical device fabrication, and multifunctional biomedical composites. Therefore, sol-gel processes are particularly suited for applications requiring structural integration and composite formation, although their limitations in controlling nanoparticle size and surface properties restrict their use as standalone colloidal UCNP systems in biomedicine.

### 2.6 Microwave-assisted synthesis

This approach emerged as a rapid, energy-efficient, and scalable technique for preparing UCNPs and other luminescent nanomaterials, offering distinct advantages over conventional heating techniques [[73], [74], [75]]. Unlike conductive heating, which relies on thermal gradients from the vessel walls, microwave heating delivers volumetric and uniform energy distribution, accelerating nucleation and crystal growth while minimizing temperature gradients and reaction time. Typical reactions that require 12-24 h under hydrothermal conditions can be completed in minutes using microwave irradiation, often between 120 and 300°C, depending on the solvent system and reactor configuration [73]. For example, UCNPs synthesized in OA and bis(2-ethylhexyl) adipate (BEHA) mixtures reached 300°C in under 5 min, with heat increments reaching 60 °C/min, allowing accurate control over particle size, phase, and shape [75]. Microwave-assisted synthesis has been successfully applied to produce NaYF_4_:Yb^3+^/Er^3+^ or Tm^3+^ UCNPs with cubic or hexagonal phases, depending on reaction time, ligand concentration, and solvent dielectric properties [74]. The resulting nanoparticles are generally small (10-50 nm), highly crystalline, and dispersible in water when hydrophilic ligands such as citrate, PEG, or PVP are incorporated during synthesis [73]. This method effectively combines the advantages of hydrothermal synthesis, such as aqueous dispersibility and biocompatibility, with vastly reduced reaction times and enhanced reproducibility. Moreover, microwave heating enables fine-tuning of crystallographic phase (e.g., cubic-to-hexagonal transition) and luminescence features, which are important factors for biomedical applications [[73], [74], [75]]. However, various limitations persist. The method requires specialized microwave reactors with precise temperature and pressure control, and the outcome is highly sensitive to the dielectric properties of solvents and precursors. Solvents must efficiently absorb microwave energy (i.e., have high dielectric loss tangent, tan δ) to ensure rapid and uniform heating. Non-polar solvents like ODE are poor microwave absorbers, whereas polar esters like BEHA offer better performance [75]. Despite these challenges, microwave-synthesized UCNPs have been successfully employed in rapid biomedical imaging, cellular labeling, and photocatalytic platforms, where their small size, high dispersibility, and optical brightness enable efficient cellular uptake and real-time tracking. Consequently, microwave-assisted synthesis provides a compelling balance between reaction speed, crystallinity, and aqueous compatibility, making it particularly attractive for rapid and scalable production of UCNPs for biomedical applications.

### 2.7 Combustion and flame spray pyrolysis

Combustion synthesis and flame spray pyrolysis (FSP) are high-temperature gas-phase techniques capable of producing large quantities of UCNPs in a rapid and scalable manner. In combustion synthesis, an exothermic redox reaction is triggered between lanthanide precursors (e.g., nitrates or acetates) and organic fuels such as glycine, urea, or citrate, resulting in the formation of nanocrystalline oxide or fluoride materials [76]. FSP, on the other hand, involves the atomization of precursor solutions into fine droplets that are combusted in a flame, typically fueled by oxygen-methane or oxygen-ethanol mixtures. This process enables continuous production of UCNP powders with tunable composition and morphology [77]. Flame-made UCNPs such as Y_2_O_3_:Yb^3+^/Er^3+^ and NaYF_4_-based systems have been synthesized with multicolor emission, and their luminescence properties can be regulated by adapting flame temperature, precursor concentration, and dopant ratios [77,78]. These methods are attractive for industrial scalability due to their simplicity, high throughput, and ability to produce kilogram-scale batches of nanoparticles in a single-step process [78]. However, the resulting products often exhibit broad size distributions, mixed phases, and surface defects, necessitating extensive post-processing such as annealing, surface modification, or sieving to improve luminescence and dispersibility [79]. While less commonly used in biomedical applications compared to colloidal methods, there are emerging examples of FSP-derived UCNPs being incorporated into polymeric composites and hybrid platforms for PDT, luminescent scaffolds, and optical biosensing [76,80]. Their robustness, thermal stability, and bulk production potential make them promising candidates for applications where material quantity, mechanical integration, or device fabrication are prioritized over ultra-high luminescence efficiency. Thus, combustion-based methods offer exceptional scalability and material throughput, although their limited control over particle uniformity and surface characteristics constrains their direct applicability in precision biomedical applications.

### 2.8 Green and bio-inspired approaches

With increasing emphasis on sustainability and biocompatibility, “green” routes for UCNP synthesis focus on aqueous media, reduced use of toxic solvents, and the incorporation of benign templating or capping agents to yield water‐dispersible materials. Water-based hydro/solvothermal protocols employing hydrophilic ligands (e.g., citric acid, PEG, PVP) are frequently highlighted as greener alternatives to high-boiling nonpolar systems and have been widely adopted in UCNP imaging and therapy workflows [66]. Recent comprehensive reviews also underscore sustainability trends in rare-earth luminescent materials, including solvent selection, ligand choice, and post-synthetic processing that minimize environmental impact while preserving performance [45].

Ionic liquids (ILs) are a prominent “green-leaning” strategy due to their negligible vapor pressure, tunable polarity, and strong microwave coupling, enabling controlled nucleation and growth at lower apparent environmental cost than volatile organic solvents. IL-assisted syntheses have produced sub-10 nm β-NaGdF_4_:Yb,Er UCNPs with enhanced crystallinity and upconversion, demonstrating that ILs can support high phase purity and small size in rare-earth fluoride hosts [81]. Earlier IL-based routes achieved spherical NaYF_4_ nanoclusters with multicolor upconversion, and subsequent IL two-phase methods extended to fluoride-oxide upconversion phosphors, highlighting the versatility of IL media for tailoring morphology and luminescence [82,83].

Bio-inspired templating and in situ hydrophilic capping, using biopolymers such as polysaccharides, are attractive for generating UCNPs with immediate aqueous dispersibility and reduced cytotoxicity. Reviews of UCNP synthesis for imaging and therapy describe the use of benign ligands and polymer coatings introduced during or immediately after nucleation to achieve colloidal stability and bioconjugation readiness, reducing or eliminating harsh ligand exchanges downstream [66]. In vivo feasibility of “green-prepared” UCNPs has been illustrated by animal studies employing water-dispersible NaYF_4_:Yb,Tm microphosphors for blue upconversion under NIR excitation, demonstrating biocompatibility-oriented synthesis and deployment in biological settings [84].

While green and bio-inspired approaches can reduce environmental burden and simplify biological integration, they often face trade-offs in size uniformity, phase control, and peak luminescence compared with thermal decomposition in nonpolar media. Reported limitations include broader size distributions and the occasional prevalence of cubic phases, necessitating careful optimization of ligands, pH, and thermal treatments to reach β-phase dominance and higher quantum yields [66]. Nonetheless, these routes are increasingly used in biosensing and imaging contexts where low cytotoxicity, direct water dispersibility, and facile functionalization are paramount, and they represent a growing direction for translational UCNP research [45,66]. Accordingly, green and bio-inspired synthesis strategies are particularly advantageous for applications prioritizing biocompatibility and environmental sustainability, although careful optimization is required to balance these benefits with optical performance.

The evolution of UCNP synthesis methods reflects a balance between the need for high luminescence efficiency, precise control over particle characteristics, scalability, and biocompatibility. Thermal decomposition remains the method of choice for producing the most efficient and monodisperse UCNPs, yet its reliance on high-temperature organic solvents and hydrophobic ligands poses obstacles for direct biomedical use. Hydrothermal and co-precipitation methods, while sometimes less efficient, are simpler, more scalable, and more directly aligned with biocompatibility requirements. Emerging approaches such as microwave-assisted synthesis, FSP, and green chemistry strategies promise to combine efficiency with scalability and sustainability. Future progress is expected to focus on three fronts. First, improving the quantum yield of UCNPs under biologically safe excitation power densities is essential for clinical translation. Second, the synthesis of ultrasmall UCNPs (<10 nm) that can be cleared renally without long-term accumulation in the body remains a critical challenge. Third, the adoption of green and scalable synthetic strategies will help forward laboratory research towards large-scale biomedical deployment. These synthesis strategies establish the fundamental structural and surface characteristics that govern upconversion mechanisms, colloidal behavior, and biological interactions, thereby forming the basis for the performance considerations discussed in the following sections.

### 2.9 Comparative analysis of synthesis strategies

The synthesis of UCNPs involves inherent trade-offs between crystallinity, particle size control, surface chemistry, scalability, and environmental impact. As demonstrated in the preceding sections, no single synthetic approach simultaneously satisfies all the requirements for optimal biomedical performance. Instead, each method provides distinct advantages that must be carefully matched to the intended application, particularly when considering factors such as luminescence efficiency, aqueous dispersibility, and suitability for surface functionalization [66,85].

Thermal decomposition remains the most effective method for producing highly crystalline and monodisperse UCNPs with superior UCL efficiency due to its precise control over nucleation and growth processes [66,86]. However, its reliance on hydrophobic ligands and high-boiling organic solvents necessitates additional post-synthetic surface modification steps to render particles water-dispersible, which may introduce variability and complexity [86]. In contrast, hydrothermal and co-precipitation approaches offer improved hydrophilicity and scalability, as they are typically performed in aqueous environments under relatively milder conditions, making them more directly compatible with biological systems [86,87]. Nevertheless, these methods often produce particles with broader size distributions and comparatively lower crystallinity, which can reduce luminescence efficiency [86]. Similarly, sol-gel and combustion-based methods provide advantages in structural integration and large-scale production, respectively, but are less suited for producing well-defined, monodisperse colloidal nanoparticles required for precision biomedical applications [66,86].

Emerging strategies such as microwave-assisted synthesis and green/ionic-liquid-based approaches aim to bridge these gaps by combining rapid processing, improved energy efficiency, and reduced environmental impact [86,88]. Microwave-assisted methods, for example, enable fast and uniform heating, leading to relatively high crystallinity within shorter reaction times, while green synthesis routes reduce reliance on toxic organic solvents [86,88]. However, these approaches still face challenges in achieving consistent control over particle size, phase purity, and luminescence performance comparable to thermal decomposition [66,86]. To provide a clearer comparison of these synthesis routes, Table 2 summarizes the key characteristics of each method in relation to parameters critical for biomedical applications.

**Table 2:** Comparative analysis of UCNP synthesis strategies and their relevance to biomedical applications.

Synthesis Method	Size Control	Crystallinity	Surface Properties	Luminescence Efficiency	Scalability	Environmental Impact	Biomedical Suitability
**Solid-state synthesis**	Limited nanoscale control [89]	High [66]	Poor dispersibility	Moderate (bulk-dominated systems)	High	Low (high temperature, energy-intensive)	Low
**Thermal decomposition**	Excellent control via nucleation-growth mechanisms [89]	High [66]	Hydrophobic (requires surface modification) [66]	High (widely used for optical applications) [66]	Moderate	Low-moderate	High after modification
**Hydrothermal /solvothermal**	Good control depending on reaction conditions [89]	High crystallinity achievable [88]	Often hydrophilic (aqueous synthesis)	Moderate-high [88]	High	Moderate (aqueous system)	High
**Co-precipitation**	Moderate control [89]	Moderate	Hydrophilic	Moderate	High	Moderate-high (simple process)	Moderate-high
**Sol-gel process**	Moderate	Moderate	Easily functionalizable matrices [89]	Moderate	High	Moderate	Moderate
**Microwave-assisted synthesis**	Good (rapid, uniform heating) [88]	High [88]	Tunable	Moderate-high [88]	High	High (energy-efficient, short time)	High (emerging)
**Combustion /FSP**	Limited control [89]	High	Limited control	Moderate	Very high	Low (high temperature, emissions)	Low-moderate
**Green /bio-inspired**	Less uniform [89]	Moderate	Biocompatible surfaces	Moderate	Moderate	Very high (eco-friendly)	High (biocompatibility advantage)

This comparative perspective highlights that the selection of a synthesis strategy is inherently application-driven. For high-resolution imaging and sensing, where luminescence efficiency and uniformity are critical, thermal decomposition remains the preferred method. Conversely, for applications emphasizing biocompatibility, scalability, and direct aqueous use, hydrothermal, co-precipitation, and green synthesis approaches offer distinct advantages. Future progress in UCNP synthesis is therefore expected to focus on hybrid strategies that integrate the optical performance of high-temperature methods with the environmental compatibility and scalability of solution-based approaches.

## 3 Basic concept and mechanism of photon upconversion

Unlike conventional fluorescence mechanisms where emitted photons carry less energy than the excitation source (Stokes emission) [3,17], photon upconversion involves a nonlinear process that results in anti-Stokes emission. This occurs when two or more photons of lower energy are absorbed sequentially, leading to the release of a single photon with higher energy. Such a mechanism is particularly valuable in biomedical imaging, as it enables excitation within the NIR window (ranging from 650 to 950 nm) where biological tissues exhibit minimal absorption and scattering [4,17]. The resulting emission, typically in the visible or ultraviolet range, offers enhanced imaging depth and clarity. UCNPs leverage this principle to achieve superior contrast and sensitivity, outperforming conventional fluorophores by reducing background autofluorescence and enabling deeper tissue visualization. In UCNPs, photon upconversion occurs through the f-f electronic transitions of lanthanide ions doped into crystalline host lattices. These ions, such as Er^3+^, Yb^3+^, Ho^3+^, and Tm^3+^, possess a unique ladder-like configuration of 4f energy levels, which are partially protected by filled 5s and 5p orbitals. This shielding minimizes interaction with the host lattice and enables sharp absorption and emission bands with long lifetimes. The host lattice, usually a low-phonon-energy fluoride such as β-NaYF_4_, provides a stable environment that suppresses non-radiative relaxation and facilitates energy transfer between sensitizer and activator ions [90]. Importantly, the practical performance of UCNPs in biomedical systems is not solely determined by the presence of these upconversion pathways, but by how efficiently they can be activated under biologically safe excitation conditions and how effectively non-radiative losses are minimized.

### 3.1 Fundamental upconversion mechanisms

#### 3.1.1 Excited-state absorption (ESA)

**Fig. 1:**
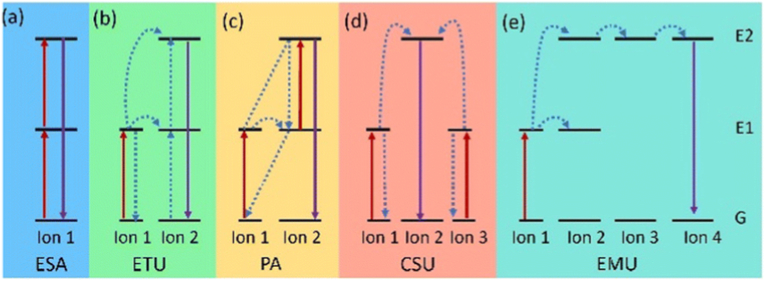
Representative upconversion pathways in lanthanide-doped UCNPs include: (a) excited-state absorption (ESA), (b) energy transfer upconversion (ETU), (c) photon avalanche (PA), (d) cooperative sensitization upconversion (CSU), and (e) energy migration-mediated upconversion (EMU). Excitation events are shown as solid red arrows, energy migration is depicted with dotted blue lines, and violet arrows indicate photon emission [91]. Copyright 2024. Adapted from the Royal Society of Chemistry. (For interpretation of the references to color in this figure legend, the reader is referred to the Web version of this article.)

This mechanism involves a single ion successively absorbing two or more photons (Fig. 1A) [91]. The ion is first excited to a metastable transient state and then further promoted to a higher-lying state upon absorbing an additional photon. From this higher state, radiative decay occurs, yielding upconverted emission. ESA is less efficient than energy transfer upconversion (ETU) in most UCNP systems because lanthanide ions have intrinsically low absorption cross-sections, but it can be significant in systems with high excitation densities [91,92]. Consequently, ESA is generally of limited practical relevance in biomedical applications, where excitation power densities must remain low to avoid photothermal damage.

#### 3.1.2 Energy transfer upconversion (ETU)

ETU is the most common mechanism (Fig. 1B) [91], which occurs when a sensitizer ion (typically Yb^3+^) absorbs a NIR photon and translocates the energy non-radiatively to an adjacent activator ion (such as Er^3+^ or Tm^3+^). Repeated energy transfer events promote the activator to higher excited states, from which it can decay radiatively, producing visible emission. ETU is particularly efficient because Yb^3+^ has a wide absorption range at 980 nm, and its energy levels match well with those of common activators [10,16]. As a result, ETU-based architectures form the foundation of most UCNP systems used in biomedical imaging, sensing, and therapy due to their relatively high efficiency under NIR excitation.

#### 3.1.3 Cooperative sensitization upconversion (CSU) and photon avalanche (PA)

Less commonly, PA and CSU processes contribute to emission (Fig. 1C and D) [91]. In CSU, two sensitizer ions simultaneously transfer energy to an activator, exciting it directly to a higher-lying state. This process requires precise spatial arrangements of dopants and is generally weaker than ETU. Photon avalanche, by contrast, is a highly nonlinear process characterized by an abrupt surge in emission intensity beyond a certain excitation threshold. It relies on a feedback loop involving excited-state absorption and cross-relaxation (CR), and while intriguing, PA requires very high excitation power and is rarely exploited in biomedical contexts [91,92]. Therefore, although CSU and PA provide important insights into nonlinear optical behavior, their stringent requirements for dopant configuration and excitation intensity limit their practical implementation in biological systems.

#### 3.1.4 Energy migration-mediated upconversion (EMU)

EMU is a sophisticated mechanism that enhances upconversion efficiency by spatially separating excitation and emission centers within a multi-shell UCNP architecture (Fig. 1E) [91]. Typically, sensitizer ions (e.g., Yb^3+^) absorb NIR photons and transmit the energy to migrator ions (e.g., Gd^3+^) in an intermediate shell, which relay the excitation to activator ions (Tm^3+^, Er^3+^) in the outer shell. This migration pathway minimizes CR and surface quenching, enabling brighter and more tunable emission. EMU designs often employ inert outer layers to further suppress non-radiative losses. The modularity of EMU structures allows precise control over energy flow, making them ideal for multiplexed imaging, lifetime tuning, and deep-tissue optogenetics. However, optimizing shell thickness and dopant distribution remains critical for maximizing performance [93]. Thus, EMU architectures illustrate how nanoscale structural design can be directly leveraged to control energy transfer pathways and enhance luminescence performance under biologically relevant conditions.

### 3.2 Factors governing upconversion efficiency and performance

#### 3.2.1 Cross-relaxation and dopant concentration effect

The efficiency of upconversion processes depends strongly on the dopant concentrations and their spatial distribution [17,34]. Too low a sensitizer concentration results in poor absorption of excitation light, while excessively high concentrations can lead to energy migration and non-radiative quenching [17]. Similarly, activator concentrations must be optimized to avoid concentration quenching, which arises from CR, a non-radiative energy transfer between adjacent activator ions that dissipates excitation energy [17]. Core-shell engineering has emerged as an effective strategy to mitigate quenching, in which an inert or undoped shell is grown around a UCNP core to isolate the emitting centers from surface defects and environmental quenchers such as water molecules [17,34]. Accordingly, dopant concentration and spatial distribution act as critical design parameters that directly influence energy transfer efficiency, luminescence intensity, and susceptibility to quenching in UCNP systems.

#### 3.2.2 Choice of excitation wavelength

Another critical factor is the choice of excitation wavelength. Traditional UCNPs utilize Yb^3+^ sensitizers excited at 980 nm, but water absorbs strongly at this wavelength, leading to local heating that can damage biological tissues. To address this issue, Nd^3+^-doped UCNPs have been developed, which absorb efficiently at 808 nm, a wavelength associated with lower water absorption. These particles transfer energy from Nd^3+^ to Yb^3+^ and then to activators (Tm^3+^ or Er^3+^), enabling efficient upconversion while reducing photothermal effects, an important consideration for in vivo applications [94]. In biomedical applications, the mechanistic diversity of photon upconversion translates into practical advantages. The sharp emission bands and long lifetimes of lanthanide transitions allow multiplexed imaging and time-gated detection, while the ability to excite in the NIR window provides deep tissue penetration. The nonlinear nature of upconversion enables localized excitation with reduced photodamage, which is particularly valuable in super-resolution imaging and optogenetics. Moreover, the dependence of emission intensity on excitation power and temperature has been exploited for nanoscale sensing of chemical and physical factors, including ion concentration, temperature, and pressure [[95], [96], [97], [98]]. The unique photophysics of lanthanide ions and the interplay of different upconversion mechanisms underpin the biomedical utility of UCNPs. Understanding and controlling these mechanisms through careful selection of host lattices, dopant ions, and nanostructure design remain central to optimizing their performance in diverse biomedical applications. Importantly, the selection of excitation wavelength represents not only a photophysical consideration but also a translational constraint, as it directly impacts thermal safety, tissue penetration, and the feasibility of in vivo applications under clinically acceptable power densities.

#### 3.2.3 Brightness enhancement strategies under biologically relevant conditions

The brightness of UCNPs is fundamentally governed by the efficiency of light absorption, energy transfer, and radiative emission processes within the nanoparticle. Due to the intrinsically low absorption cross-sections of lanthanide ions and the susceptibility of excited states to non-radiative relaxation, enhancing emission efficiency requires careful control over photophysical pathways at the nanoscale. One of the most effective approaches involves core-shell and multishell engineering, where inert or active shells are introduced to suppress surface-related quenching and regulate energy migration. Inert shells physically isolate emitting centers from surface defects and high-energy vibrational modes, thereby reducing non-radiative decay. Active shells, on the other hand, can be designed to facilitate directional energy transfer or broaden excitation profiles. Multilayer architectures incorporating sensitizer, migrator, and activator ions enable spatial separation of excitation and emission processes, as described in EMU, leading to improved radiative efficiency and tunable emission characteristics [99]. Sensitization engineering provides another key route to enhance excitation efficiency. Nd^3+^-based sensitization enables excitation at 808 nm, offering an alternative to conventional Yb^3+^-based systems excited at 980 nm. This approach improves light absorption in biologically relevant spectral windows while maintaining efficient energy transfer to activator ions, although it requires careful optimization to minimize energy loss through competing pathways. To overcome the limitations of lanthanide absorption, dye-sensitized UCNPs have been developed, where organic chromophores act as antennae to harvest excitation light and transfer energy to the lanthanide system [100]. This strategy significantly enhances absorption efficiency but introduces challenges related to photobleaching, stability, and interfacial coupling between dye molecules and the nanoparticle surface.

Plasmonic coupling has also been explored as a means to enhance local electromagnetic fields and increase excitation or emission rates. Hybrid systems incorporating metallic nanostructures such as gold or silver nanoparticles can lead to substantial luminescence enhancement [101]. However, these effects depend strongly on spatial configuration and spectral overlap, and may introduce additional non-radiative losses or structural complexity. Brightness enhancement at the photophysical level relies on optimizing energy transfer efficiency, minimizing non-radiative pathways, and improving excitation absorption within the nanoparticle architecture. These strategies establish the intrinsic limits of UCNP emission performance, which are further modulated by material properties and environmental conditions discussed in the following sections.

## 4 Engineering the bio-nano interface: from structural design to biological performance

The biomedical applicability of UCNPs depends not only on their fundamental luminescent features but also on a broader set of physicochemical properties, including colloidal stability, optical tunability, and targeted biological interactions. These properties do not operate independently; rather, they collectively determine the performance of UCNPs in biological system. In the following subsections, we explore how rational surface and structural engineering strategies (ranging from polymeric coatings to complex core-shell architectures) are deployed to overcome the biological and physical determinants that limit UCNP performance.

### 4.1 Colloidal stability and polymeric/biomimetic coatings

Colloidal stability is a prerequisite for the biomedical use of UCNPs, as aggregation in biological fluids can lead to loss of optical function, altered biodistribution, reduced targeting accuracy, and increased cytotoxicity [17,102]. As-synthesized UCNPs are often capped with hydrophobic ligands such as OA, which preserve dispersion in nonpolar solvents but render the nanoparticles poorly compatible with aqueous and physiological environments [17]. Consequently, the transition from synthetic media to biological systems introduces a fundamental interfacial challenge. At the system level, colloidal stability in biological media is governed not only by electrostatic or steric repulsion but also by the dynamic interactions of UCNPs with salts, proteins, lipids, and other biomolecules in serum and extracellular fluids. These interactions can promote aggregation, alter surface charge, and generate adsorbed biomolecular layers (the protein corona) that redefine the effective biological identity of the nanoparticle.

**Fig. 2:**
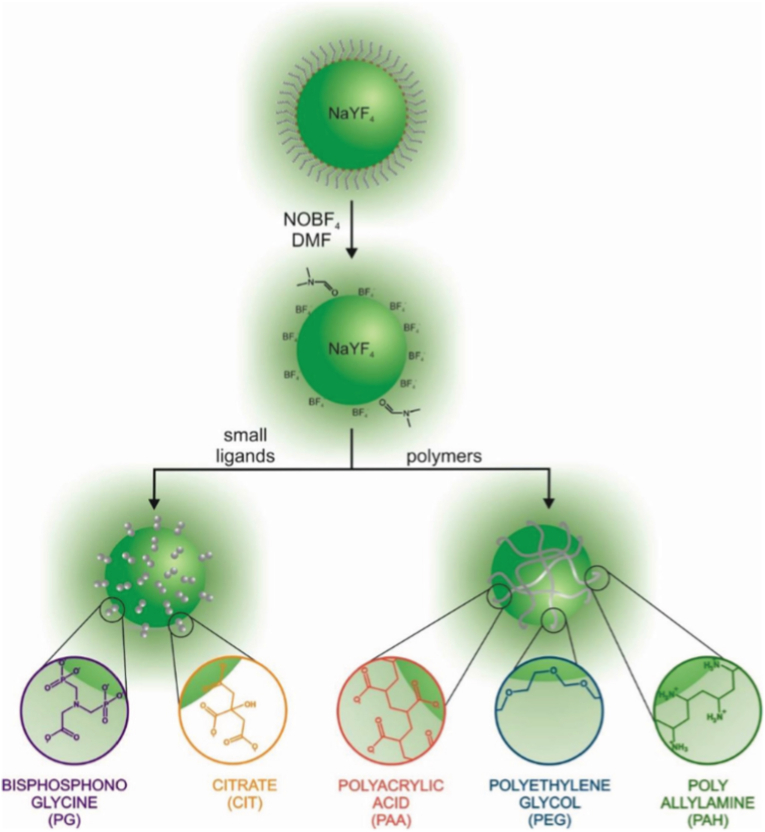
Two-step ligand exchange strategy for UCNP surface modification: oleate is first removed using NOBF_4_, yielding BF_4_^−^-stabilized bare particles, followed by functionalization with ligands such as PG, CIT, PAA, PEG, or PAH [103]. Copyright 2019. Adapted from John Wiley & Sons.

While simple hydrophilic surface modifications, such as carboxyl-functionalization via malonic acid [102] or basic PEG/PVP ligand exchanges [17], can achieve baseline water dispersibility, these approaches often fall short in high-ionic-strength or protein-rich environments. For instance, Himmelstoβ et al. demonstrated through a comprehensive comparison of surface stabilization that while small-molecule ligands (citrate, phosphonoglycine) fail under physiological ionic strength, polymer-coated systems (PEG, PAH, PAA) successfully resist dilution-induced disintegration and aggregation even at 2.5 M NaCl (Fig. 2) [103]. Therefore, polymeric and biomimetic coatings have emerged not merely as solubilizing layers, but as functional interfacial architectures that actively regulate how UCNPs interact with complex biological environments over time.

Among polymeric coatings, PEG remains the most extensively employed due to its hydrophilicity, non-immunogenicity, and ability to reduce nonspecific protein adsorption through steric hindrance. However, recent studies emphasize that polymer coatings are not passive barriers, but active determinants of biological interaction pathways that dictate the specific evolution of the protein corona. For example, PIMA-PEG and PMAO-PEG copolymers have shown remarkable capacity to maintain UCNP dispersion for over three months in PBS, but more importantly, different polymer architectures generated distinct protein adsorption profiles that subsequently altered the time-dependent cellular internalization from clathrin- and caveolae-independent pathways toward clathrin-mediated uptake [104]. This highlights a recurring translational trade-off: while dense PEG-based coatings successfully reduce macrophage uptake to less than 1.0% and improve stealth behavior, they may simultaneously hinder internalization by target tumor cells [104]. To further enhance chemical robustness, polymers like PEG-alendronate (PEG-Ale) have been employed to significantly delay fluoride release and preserve cell viability, demonstrating that polymers protect both the colloidal state and the intrinsic crystal structure [105].

Beyond passive stabilization, polymers can couple stability with direct chemical functionality. PAA coatings provide a prime example. In one system, PAA-modified UCNPs acted as Cu^2+^ sensors where the surface carboxylates participated directly in analyte binding, leading to linear and reversible fluorescence quenching [106]. In diagnostic settings, optimized PAA grafting has enabled stable, single-particle detection in complex plasma for thyroid-stimulating hormone immunoassays, proving that polymers can act as molecularly active scaffolds without compromising dispersion [107].

**Fig. 3:**
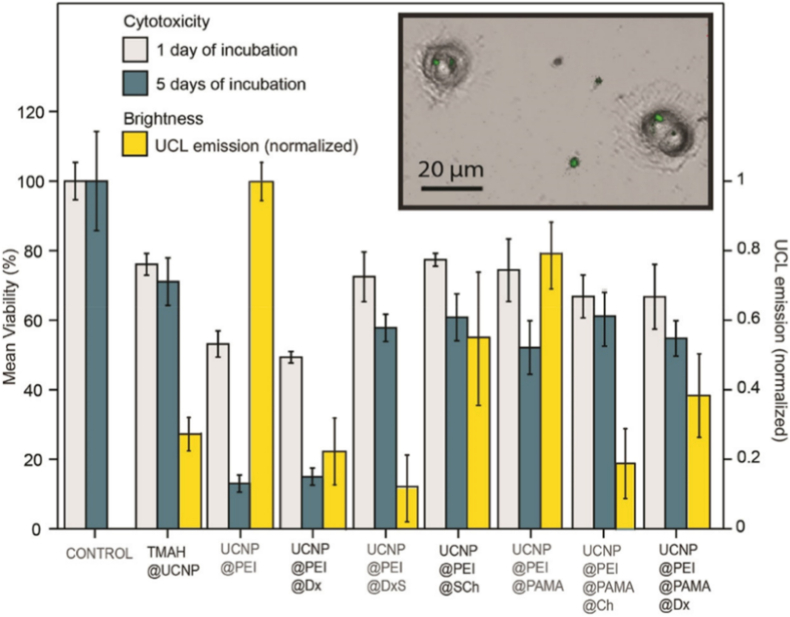
Cytotoxicity and brightness of UCNP@PEI and its coated variants: gray and blue bars show HaCaT cell viability after 24 h and 120 h exposure (125 μg/mL), while yellow bars indicate relative UCL brightness in water. Inset: Confocal image of UCNP@PEI uptake by HaCaT cells after 48 h, with green UCL marking internalized and external particles [109]. Copyright 2018. Adapted from the American Chemical Society. (For interpretation of the references to color in this figure legend, the reader is referred to the Web version of this article.)

Other polymer systems highlight the tension between optical enhancement, functionality, and biocompatibility. Polymethyl methacrylate (PMMA), for instance, serves as an excellent optically transparent matrix that, when combined with plasmonic nanoparticles, can amplify local electric fields and enhance emission up to 150 times [108]. Conversely, polyethylenimine (PEI) is highly attractive because its dense amine groups shield water-induced quenching (yielding the highest UCL intensity among tested formulations) and promote rapid cellular uptake [109]. However, this brightness comes at the cost of severe cytotoxicity, reducing cell viability by over 85% after 120 h. Mitigating this toxicity requires secondary protective coatings (e.g., chitosan or dextran), underscoring the delicate balance required in bio-nano interface design (Fig. 3) [109].

To circumvent the limitations of synthetic polymers, biomimetic and zwitterionic surface chemistries offer advanced interfacial stabilization. Zwitterionic phosphorylcholine-based polymers (PMPC) enhance in vivo circulation by mimicking cell-membrane behaviors and resisting biofouling [110]. Similarly, micellar structures utilizing F127-TPGS have shown robust in vivo circulation stability critical for tumor imaging [35]. Ultimately, the engineering of the UCNP surface must move beyond the simple goal of water solubility. Polymeric and biomimetic coatings must be viewed as dynamic interfacial regulators whose translational success relies entirely on their ability to resist nonspecific adsorption, control the protein corona, modulate immune recognition, and maintain functional optical accessibility under realistic in vivo conditions.

### 4.2 Optical design, brightness, and core-shell/silica architectures

The hallmark of UCNPs is their optical performance, specifically, their ability to convert low-energy NIR excitation into visible or ultraviolet emission. However, the observable brightness and functionality of UCNPs in biomedical applications are strongly influenced by the interplay between material design and environmental conditions. A fundamental determinant of optical performance is the selection of dopant ions and the host lattice. For instance, Kang et al. demonstrated that the crystal phase of NaYF_4_:Yb^3+^/Er^3+^ UCNPs plays a critical role, with the cubic α-phase exhibiting enhanced red emission compared to hexagonal β-phase systems under specific conditions [10]. Similarly, spectral tunability can be engineered through dopant combinations; Yb^3+^/Ho^3+^-doped systems produce red emission favorable for deep tissue imaging due to reduced scattering [24], while precise synthesis tuning (e.g., reaction temperatures) allows the modulation of red-to-green emission ratios vital for imaging contrast [111].

While intrinsic efficiency is governed by these compositional choices, UCNP brightness in biological environments is severely limited by surface-related quenching from water molecules and surface defects. Core-shell and multilayer architectures represent a critical structural strategy to overcome these photophysical limits. By encapsulating a luminescent core within an inert or active shell, these structures physically isolate emitting centers from environmental quenchers. Zhou et al. demonstrated this by engineering NaYF_4_:Yb,Er@NaYF_4_ core-shell UCNPs that showed a five-fold increase in emission intensity compared to bare cores, attributed entirely to reduced non-radiative relaxation at the particle surface [34]. Furthermore, the microsecond-to-millisecond emission lifetimes characteristic of these protected lanthanide transitions enable time-gated detection, effectively suppressing short-lived background autofluorescence [34].

Beyond inert protection, active shell engineering allows for the deliberate manipulation of excitation pathways, which is critical for safe biomedical use. Traditional Yb^3+^-sensitized UCNPs rely on 980 nm excitation, a wavelength strongly absorbed by water, leading to local tissue heating. To address this, core-shell designs incorporating Nd^3+^ have been developed to shift excitation to the 808 nm window, significantly reducing photothermal damage. For example, Du et al. reported efficient energy transfer from Nd^3+^ to Yb^3+^ in a multi-shell NaLuF_4_ system under 808 nm excitation [4], while Liu et al. demonstrated that Nd^3+^/Yb^3+^/Er^3+^ co-doped UCNPs minimized photothermal effects in live mice, representing a significant advance for safe in vivo imaging [39]. In therapeutic applications, coating a NaYF_4_ core with a NaYF_4_:Nd^3+^ shell has not only shifted excitation to 800 nm but also enabled robust conjugation with photosensitizers like hypericin to achieve potent PDT effects [112].

While fluoride-based shells optimize photophysics, silica and mesoporous shells provide the structural versatility needed to bridge optical performance with biological utility. Silica encapsulation, often achieved via reverse microemulsion or Stöber methods, is chemically inert, optically transparent, and provides a highly functionalizable surface (-OH and -NH_2_ groups) that stabilizes UCNPs while preserving imaging contrast [113,114]. More importantly, these shells can be engineered with distinct porosities. Mesoporous silica shells offer an architecture that can be exploited for drug loading, such as rattle-structured organo-silica-shelled UCNPs developed for simultaneous imaging and therapy via co-loaded photosensitizers [115].

**Fig. 4:**
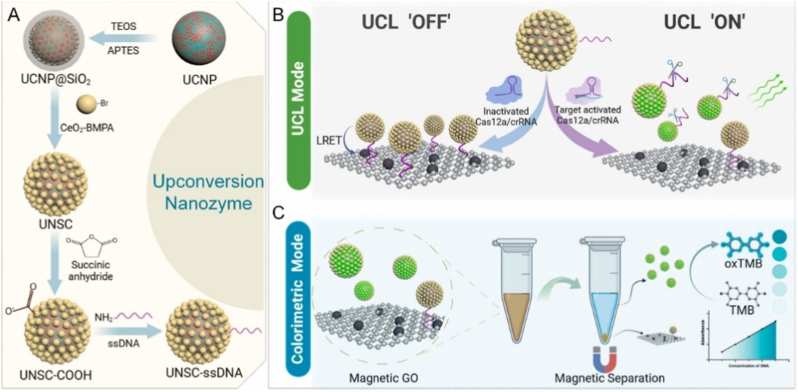
Design of UCNP@SiO_2_/CeO_2_ nanocomposites for CRISPR-Cas12a dual-mode biosensing. **(A)** Core-satellite UNSC structure featuring UCNPs at the center and CeO_2_ nanodots on the silica shell. **(B)** UCL assay mechanism. **(C)** Colorimetric assay mechanism [11]. Copyright 2025. Reproduced with permission from Elsevier.

The most advanced bio-nano interfaces combine these strategies into highly complex, multilayered composites. Lu et al. employed a NaGdF4 core, coated it with an active NaGdF_4_ shell, further encapsulated it in silica (SiO_2_), and finally modified it with PEG and trimethyl chitosan (TMC) [116]. This multilayered design not only improved luminescence and colloidal stability but also significantly enhanced adsorptive-mediated transcytosis across human endothelial barriers [116]. Similarly, Yan et al. integrated UCNP cores with silica shells and CeO_2_ nanodots (UCNPs functions as luminescence donors in a LRET-based assay) to create a CRISPR-Cas12a dual-mode biosensing platform, proving that structural hierarchy directly enables advanced bioanalytical tasks (Fig. 4) [11].

From a critical perspective, while core-shell and silica architectures drastically improve luminescence and enable multifunctionality, they introduce a distinct translational challenge. The addition of multiple epitaxial fluoride layers or porous silica shells significantly increases synthetic complexity, leading to difficulties in batch-to-batch reproducibility. Furthermore, while silica facilitates bioconjugation, its inherent surface hydroxyl groups are known high-energy oscillators that can inadvertently quench luminescence if the core is not adequately protected by a sufficiently thick inert inner shell. Therefore, the architectural design of UCNPs must be viewed as a careful compromise: researchers must balance the need for high optical yield and multifunctional loading capacity against the practical requirements of synthetic scalability, overall particle size limits for biological clearance, and in vivo stability.

### 4.3 Bioconjugation and stimuli-responsive interfaces

The simplest and most direct approach to preparing UCNPs for targeted biological interactions is surface conversion via ligand exchange or small-molecule functionalization. In this process, the native hydrophobic ligands (such as oleate) are replaced with hydrophilic molecules bearing reactive terminal groups. For example, Wang et al. demonstrated the exchange of OA ligands on NaYF_4_:Yb,Tm UCNPs with PAA, producing carboxyl-functionalized nanoparticles that were stably dispersed in aqueous media and enabled DNA conjugation [117]. Similarly, Liebherr et al. reported ligand exchange with maleimide-PEG, which introduced carboxyl groups for anchoring and exposed thiol-reactive maleimide moieties, allowing efficient protein coupling [118]. In addition to exchange, direct ligand oxidation has been utilized; Chen et al. oxidized oleate to azelaic acid, exposing terminal carboxyl groups [119], though subsequent studies highlighted the drawbacks of prolonged oxidation and the need for emission recovery strategies [120,121]. While these small-molecule approaches are advantageous for preserving particle size, they may lead to partial ligand detachment or incomplete coverage, necessitating the transition to more robust covalent bioconjugation strategies.

Beyond simple stabilization, surface functionalization provides the chemical handles (carboxyl, amine, and thiol groups) required for the covalent bioconjugation of biomolecules, such as peptides, antibodies, enzymes, nucleic acids, or aptamers, to achieve high biological specificity. PEGylated UCNPs are widely employed in this regard. Kostiv et al. demonstrated versatile conjugation strategies using heterobifunctional PEG linkers bearing neridronate and alkyne or maleimide groups, enabling efficient click chemistry with azide-modified streptavidin or antibodies. This yielded UCNP bioconjugates with high colloidal stability and low detection limits in upconversion-linked immunosorbent assays (ULISA) [122]. Makhneva et al. further optimized UCNP-antibody conjugates for both heterogeneous and homogeneous immunoassays targeting tumor protein p53 and prostate-specific antigen (PSA), achieving single-molecule recognition without washing steps [123]. In targeted delivery, Yadav et al. utilized folic acid conjugation on UCNP-loaded micelles to enable selective uptake by FRα-positive lung cancer cells, validating the role of bioconjugation in improving therapeutic index and biodistribution [35].

**Fig. 5:**
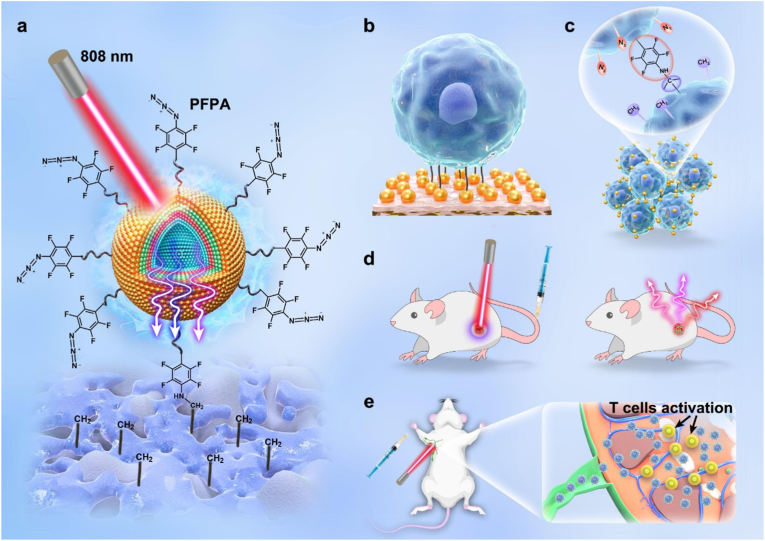
LINC enables precise bioconjugation via NIR-triggered activation of PFPA-modified UCNPs: (**a**) schematic of the mechanism; (**b-e**) demonstrations of in vitro cell-material bonding, cell-cell fusion for osteoclast formation, and in vivo targeting of tumors and lymph nodes [124]. Copyright 2024. Reproduced with permission from Elsevier.

**Fig. 6:**
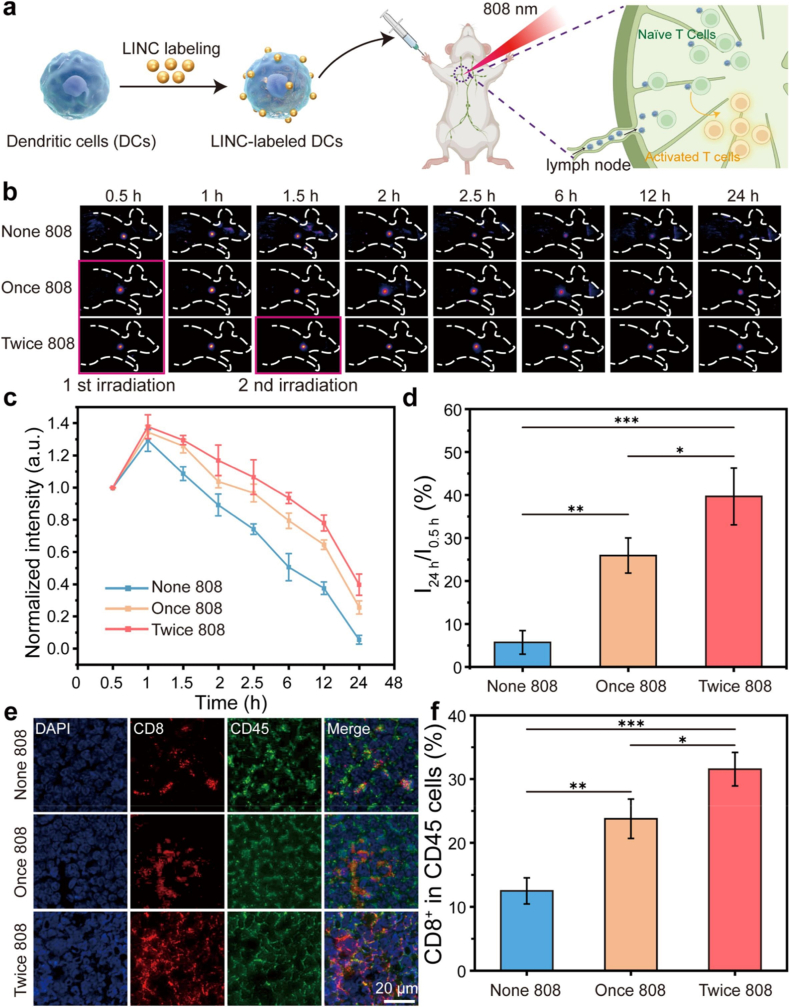
LINC-enabled lymph node targeting and immune activation in vivo. (a) Schematic of LINC-labeled dendritic cells migrating to lymph nodes and undergoing light-triggered bioconjugation under repeated 808 nm irradiation. (b) Time-dependent NIR imaging of mice across treatment groups (no irradiation, single, and repeated irradiation). (c) Normalized luminescence intensity profiles at lymph nodes over time. (d) Ratio of signal retention between 24 h and 0.5 h post-injection. (e) Immunofluorescence analysis of CD8 and CD45 expression in lymph node sections (DAPI for nuclei). (f) Quantification of CD8^+^ cells within CD45^+^ populations [124]. Copyright 2024. Reproduced with permission from Elsevier.

Recent advances have moved beyond static bioconjugation toward spatiotemporally controlled, stimuli-responsive interfaces using NIR light. Hu et al. developed a NIR light-initiated nano-crosslinker (LINC) by integrating UV-emissive upconversion nanocomposites with perfluorophenyl azide (PFPA), enabling covalent bioconjugation only under localized 808 nm irradiation [124]. The UCNP core functions as a phototransducer, converting NIR light into localized UV emission that activates PFPA to generate highly reactive nitrene intermediates (Fig. 5) [124]. These species insert into nearby C-H and N-H bonds, allowing covalent attachment without requiring pre-installed functional groups. This programmable bioconjugation was effectively exploited to modulate immune cell behavior, where LINC-labeled dendritic cells migrated to lymph nodes and were retained there through localized, NIR-triggered crosslinking, leading to increased CD8^+^ T cell populations (Fig. 6) [124].

This paradigm of dynamic control extends to interfaces that respond to chemical and physical triggers such as pH, temperature, or target analytes. One effective approach involves embedding UCNPs within mesoporous silica or metal-organic frameworks (MOFs) to create hybrid platforms capable of triggered release. Dubey et al. utilized mesoporous SiO_2_ to encapsulate both hydrophilic and hydrophobic drugs around a UCNP core, relying on the mildly acidic tumor microenvironment (pH 5.0) to preferentially release the payload while retaining imaging capabilities [125]. Taking this a step further, Abucafy et al. synthesized UCNP@ZIF-8 core-shell nanocomposites that responded to both acidic pH and 980 nm laser irradiation, achieving twin-stimuli-reactive release that maximized cytotoxicity toward MCF-7 cells with minimal off-target effects [126]. Thermal triggers have also been integrated; Gao et al. designed UCNP-poly(N-isopropylacrylamide)/DNA core-shell microgels where a thermosensitive polymer shell enabled programmable DNA release, enhanced by NIR-triggered localized heating [127].

From a critical perspective, bioconjugation and stimuli-responsive engineering successfully transform UCNPs from passive optical labels into highly specific, intelligent nanodevices. However, this functional sophistication introduces significant translational hurdles. The efficiency of stimuli-responsive release (e.g., pH or NIR-triggered) is rarely absolute; these systems often suffer from premature payload leakage during systemic circulation. Furthermore, while systems like the LINC platform showcase brilliant spatiotemporal control, they rely on upconverted UV emission. Because UV light is highly attenuated by biological tissue, the phototransduction must occur directly at the bio-nano interface, requiring extremely high local excitation densities that may not be feasible in deep-tissue clinical applications. Finally, the dense covalent tethering of antibodies or aptamers can inadvertently alter their 3D conformation, reducing binding affinity. Therefore, future designs must prioritize simpler, highly robust conjugation chemistries (such as bio-orthogonal click reactions) and aim to minimize the “moving parts” in stimuli-responsive systems to ensure predictable in vivo behavior.

**Fig. 7:**
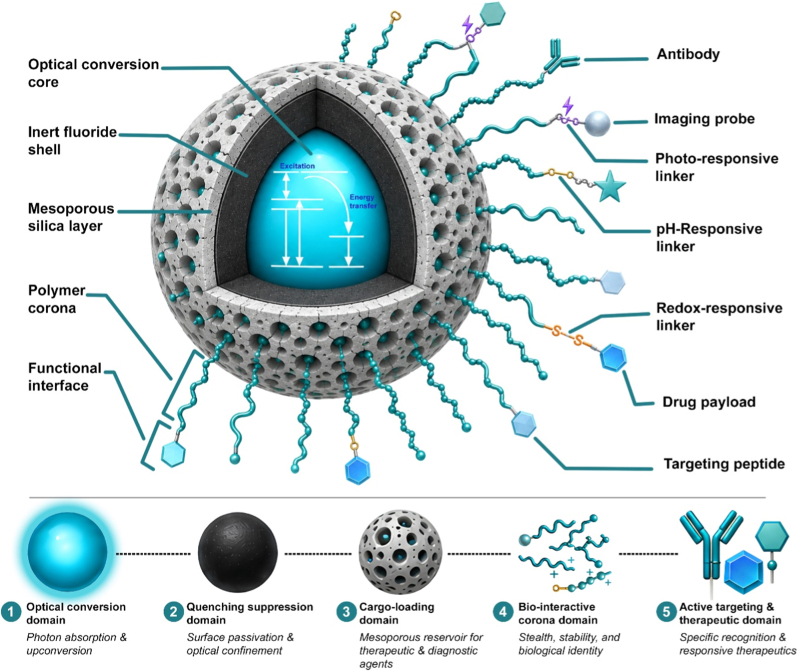
Hierarchical bio-nano interface architecture of advanced UCNP platforms. The UCNP functions as a modular, systems-level nanodevice composed of synergistically integrated domains: (1) an optical conversion core, (2) protective quenching suppression shells, (3) a mesoporous silica cargo reservoir, (4) a biointeractive polymer corona, and (5) targeting molecules. Combining optical confinement with targeted, stimuli-responsive surface engineering dictates the ultimate in vivo functionality and translational viability of the nanoplatform.

Ultimately, the successful translation of UCNPs relies on moving beyond single-variable optimizations to view the nanoparticle as a fully programmable, hierarchical nanoplatform. As summarized in (Fig. 7), this architectural paradigm divides the UCNP into distinct, synergistic domains. The innermost lanthanide-doped core and epitaxially grown fluoride shells act as the optical conversion and quenching suppression domains, strictly governing the energy transfer photophysics. Moving outward, mesoporous silica networks serve as structural cargo-loading reservoirs, while the outermost polymer corona dictates the particle's biological identity, mediating colloidal stability, stealth behavior, and the evasion of protein corona formation. By docking highly specific bioconjugates and stimuli-responsive linkers (e.g., pH, redox, or photo-cleavable bonds) onto this peripheral corona, the UCNP is transformed into an active biointerface. This modular integration of optical physics, structural chemistry, and biological engineering forms the fundamental prerequisite for deploying UCNPs in the advanced theranostic applications discussed in the following sections.

### 4.4 Multimodal functionality and hybrid integration

Building upon the optical tuning and targeted bio-interfaces discussed previously, the structural integration of non-optical functionalities extends the scope of UCNPs into multimodal theranostic platforms. While UCNPs offer exceptional optical contrast, combining them with magnetic, plasmonic, or photothermal materials overcomes the inherent depth limitations of optical imaging and enables synergistic therapeutic interventions. The most straightforward approach to multimodal integration relies on intrinsic host doping. The incorporation of paramagnetic Gd^3+^ ions is a widely explored strategy, as it contributes to energy transfer while simultaneously providing strong *T*_1_ contrast for MRI. Wang et al. described NaGdF_4_:Yb,Er nanoparticles that seamlessly combined UCL with MRI contrast, enabling dual-mode imaging of tumors in vivo without the need for complex secondary nanostructures [21]. Similarly, Dibaba et al. reported NaYF_4_:Yb,Er@NaGdF_4_ core-shell UCNPs with enhanced luminescence and robust MRI contrast, demonstrating the compatibility of Gd^3+^ shells with both optical protection and magnetic functionalities [16].

Beyond intrinsic doping, hybridization with discrete functional nanomaterials, such as superparamagnetic iron oxide nanoparticles (SPIONs) or plasmonic gold, offers a more modular, albeit complex, integration strategy. Zhang et al. synthesized SiO_2_-coated Fe_3_O_4_@NaYF_4_:Yb/Er “nanorattles” for magnetically guided chemotherapy and luminescence imaging, achieving 96% tumor reduction in vivo under combined NIR irradiation and magnetic targeting [128]. Cheng et al. developed UCNP-IONP composites modified with PEG and gold shells, which showed enhanced accumulation at tumor sites under magnetic guidance and enabled complete tumor ablation via photothermal therapy (PTT) [129]. Other hybrid systems have achieved triple-modality capabilities (UCL, down-conversion fluorescence, MRI) by embedding UCNPs, ultra-small IONPs, and organic dyes within polymeric micelles [130]. These architectures illustrate how UCNPs can be interfaced with complementary components to support synergistic therapies, such as combining PTT with magnetic hyperthermia [131].

From a critical perspective, while hybrid integration drastically expands the diagnostic and therapeutic scope of UCNPs, it introduces competing physical demands. Integrating dark, highly absorbing materials like SPIONs or plasmonic gold frequently dampens UCL due to competitive absorption of the excitation light or non-radiative energy transfer. Furthermore, assembling multiple distinct nanoparticles into a single hybrid platform inevitably increases the overall hydrodynamic diameter of the construct. As discussed in the following section, crossing the 30-50 nm size threshold fundamentally alters the biological fate of the particle, often resulting in rapid clearance by the liver and spleen before the multimodal functionalities can be exploited at the target site.

### 4.5 Biological fate, clearance, and toxicity

The biological fate of UCNPs represents the ultimate manifestation of the design principles discussed in the preceding sections. While core-shell engineering, polymeric coatings, and hybrid integration govern functionality at the material level, these exact parameters collectively dictate how UCNPs interact with complex biological environments in vivo. Understanding biocompatibility, biodistribution, and long-term retention is therefore not an isolated toxicological consideration, but a direct consequence of rational UCNP design.

Unmodified lanthanide-based nanoparticles are known to interfere with biological processes, including the inactivation of intracellular ATP, leading to oxidative stress, inflammation, and cellular damage [105,[132], [133], [134], [135], [136]]. The bio-nano interface engineering discussed in earlier sections 4.1, 4.2 plays a critical role in mitigating these effects. Inorganic shells (e.g., silica) and dense polymer coatings effectively suppress lanthanide ion leakage, preventing macrophage-associated cytotoxicity and ensuring minimal changes to serum biochemistry or organ histology even at relatively high doses [[137], [138], [139], [140]]. However, following systemic administration, the surface charge and protein corona of these engineered particles dictate their biodistribution. Positively charged UCNPs heavily adsorb serum proteins, accelerating opsonization and clearance [141]. Regardless of the coating, intravenous administration consistently results in the rapid sequestration of nanoparticles by the mononuclear phagocyte system (MPS), particularly by Kupffer cells in the liver and macrophages in the spleen, within hours of injection [[137], [138], [139]].

The subsequent pharmacokinetics and clearance pathways of UCNPs are rigidly size-dependent. Smaller UCNPs (approximately 10 nm) demonstrate partial renal excretion, presenting detectable nanoparticle signals in urine [142,143]. Conversely, the highly engineered, multifunctional UCNPs (typically >30 nm) required for optimal brightness and drug loading are entirely precluded from renal filtration. These larger particles rely on slower hepatobiliary pathways, where they are internalized by the liver and gradually excreted through feces [144,145]. Surface engineering can delay this systemic clearance; PEGylation significantly prolongs blood circulation [139,146], and biomimetic red blood cell (RBC) membranes can extend circulation half-lives to nearly 40 h by actively suppressing macrophage uptake [147]. Despite gradual clearance, the long-term retention of UCNPs remains a major translational roadblock. Elemental analyses routinely reveal stable levels of Y^3+^ in the liver and spleen for up to three months post-injection, sequestered within intracellular compartments long after optical signals diminish [137,139]. This incomplete elimination raises significant concerns regarding the bioaccumulation of heavy rare-earth elements and potential chronic toxicity.

**Fig. 8:**
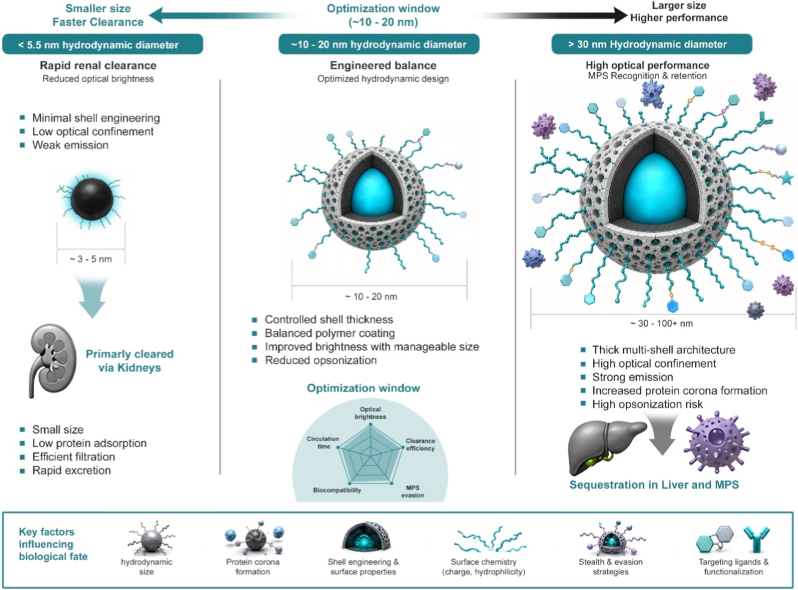
The size-clearance paradox governing the biological fate and optical performance of UCNPs. A fundamental translational trade-off exists between ultra-small particles that undergo rapid renal filtration but exhibit weak luminescence, and highly engineered UCNPs that achieve superior brightness but suffer from heavy protein corona formation and rapid MPS sequestration. Successful clinical translation requires targeting a central optimization window to finely balance parameters to achieve optimal behavior.

This fundamental optical-biological trade-off represents a central translational barrier, conceptually summarized as the size-clearance paradox (Fig. 8). At one extreme, ultra-small UCNPs (<5.5 nm hydrodynamic diameter) exhibit highly favorable rapid renal clearance and minimal protein adsorption; however, they inevitably suffer from weak emission due to severe surface quenching and a lack of protective optical confinement. Conversely, the highly engineered, multishell theranostic platforms (>30 nm) achieve brilliant luminescence and high cargo capacity but inevitably trigger pronounced protein corona formation. This dense bio-molecular layer accelerates opsonization and rapid recognition by the MPS, leading to heavy sequestration by Kupffer cells in the liver and macrophages in the spleen. Therefore, bridging these extremes requires targeting a narrow engineering optimization window (∼10-20 nm). Within this regime, stealth polymer coatings, precisely controlled inert shell thicknesses, and finely tuned surface chemistries must be perfectly balanced. The goal is to maintain sufficient optical brightness and colloidal stability while actively evading macrophage uptake and enabling safe, predictable biological clearance.

From a critically reflective standpoint, the field of UCNP design is currently caught in a fundamental size-clearance paradox. The architectural requirements for high upconversion quantum yield, robust colloidal stability, and multimodal integration generally push the hydrodynamic diameter of UCNPs well beyond the renal clearance threshold (∼5.5 nm). Consequently, the highest-performing theranostic UCNPs are the ones most likely to persist indefinitely in reticuloendothelial organs. While thick silica or polymer shells successfully mask acute toxicity, they also hinder enzymatic biodegradation [132,136]. Effective clinical translation will therefore require a paradigm shift toward intentionally biodegradable UCNP architectures [136,148,149] that provide transient structural stability for imaging and therapy, but predictably disassemble into renally clearable sub-components to prevent long-term heavy metal retention.

## 5 Biomedical applications of upconversion nanoparticles

The biomedical utility of UCNPs arises from unique photophysical properties and tunable material architectures that enable functionalities inaccessible with conventional fluorophores. Converting NIR excitation into higher-energy anti-Stokes emission minimizes tissue autofluorescence and photodamage, while sharp emission bands, long lifetimes, and photostability support high signal fidelity. However, these intrinsic advantages alone do not define performance. Instead, macroscopic biomedical utility is fundamentally governed by the microscopic photophysical pathways detailed in the preceding sections. While application-driven literature frequently omits explicit mechanical labels, the performance of these nanoplatforms remains strictly dictated by host-dopant configurations and core-shell architectures.

Different application classes consequently impose distinct photophysical design priorities. For instance, optical biosensors and therapeutic systems such as PDT heavily exploit high-efficiency ETU pathways. Typically utilizing Yb^3+^ sensitizers paired with Er^3+^ or Tm^3+^ activators, ETU translates low-energy NIR light into local ultraviolet or visible emissions required to trigger chemical assays, activate photosensitizers, or cleave photo-responsive drug carriers. Conversely, emerging smart systems, including multiplexed bioimaging, optogenetics, and logic-gated platforms, demand programmable activation and orthogonal excitation. These complex modalities increasingly rely on EMU and multi-shell isolation structures to suppress surface quenching, avoid CR bottlenecks, and tune precise emission lines. Therefore, UCNPs are not universally optimized materials, but rather application-specific nanoplatforms whose operational parameters can be systematically contextualized via their underlying upconversion mechanisms. The following sections analyze these biomedical domains, categorized into sensing and diagnostics, bioimaging, therapy and theranostics, and remote biological control, by explicitly linking UCNP design strategies and photophysical mechanisms to functional outcomes, representative advances, and current limitations.

### 5.1 Sensing and analytical diagnostics

The performance of UCNP-based sensing systems is fundamentally determined by two complementary design paradigms: intrinsic optical transduction and interfacial signal modulation. In the first, sensing arises directly from the sensitivity of lanthanide emission to environmental changes such as temperature, pressure, or local fields. In the second, UCNPs serve as optical transducers whose emission is modulated through interactions with biomolecules, quenchers, or hybrid nanostructures. These two approaches correspond to distinct material requirements. Intrinsic sensing depends primarily on host lattice composition, dopant configuration, and suppression of nonradiative pathways, whereas biosensing and analytical diagnostics rely more heavily on surface functionalization, donor-acceptor coupling, and nanoscale control of interparticle interactions.

#### 5.1.1 Intrinsic optical transduction for physical sensing

Physical sensing applications illustrate how UCNPs can directly convert environmental perturbations into measurable optical signals without requiring external recognition elements. In these systems, the sensing mechanism is embedded within the photophysics of the material itself, making lattice design and energy transfer pathways the dominant performance determinants. A representative example is the mechanosensitive UCNP system developed by McLellan et al., where Yb^3+^/Er^3+^-doped particles in alkaline-earth rare-earth host lattices exhibit pressure-dependent modulation of red-to-green emission ratios [150]. This behavior directly exploits the ETU pathways, where pressure-induced changes in the crystal field alter nonradiative relaxation rates and intra-ionic energy transfer dynamics. The resulting ratiometric response enables quantitative mechanical sensing at the nanoscale, demonstrating how emission characteristics can directly encode physical stimuli.

**Fig. 9:**
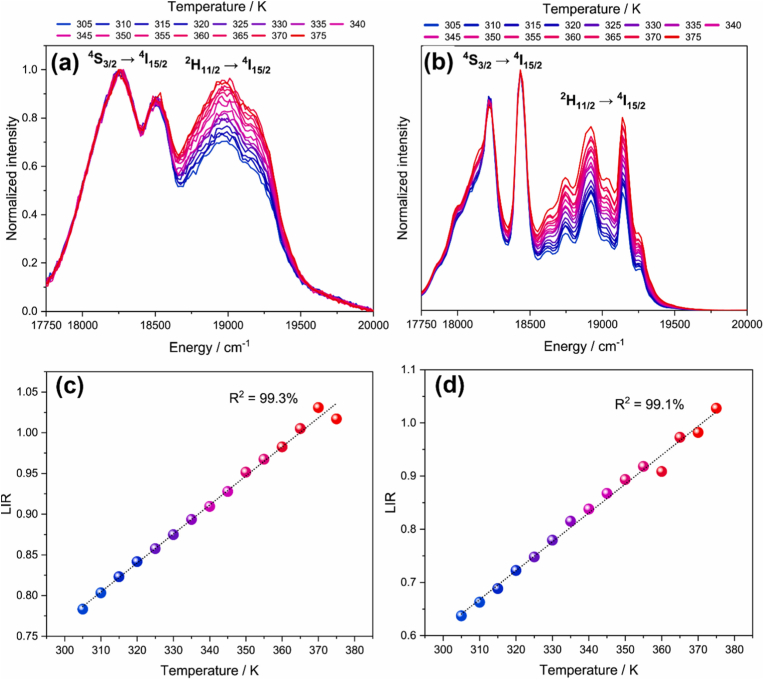
(**a-b**) Temperature-dependent UC spectra under 808 and 980 nm excitation. (**c-d**) Linear LIR response and thermal sensitivity plots, confirming suitability for ratiometric nanothermometry [33]. Copyright 2024. Reproduced with permission from Elsevier.

Thermometry provides another well-established example of intrinsic UCNP sensing. Systems based on thermally coupled Er^3+^ levels (^2^H_11_/_2_ and ^4^S_3_/_2_) generate temperature-dependent emission ratios governed by Boltzmann statistics. While early implementations relied on core-only particles, recent advances demonstrate that performance is strongly enhanced through structural engineering. Garcia et al. showed that both inert shell passivation and active shell doping significantly improve emission intensity, lifetime, and thermal sensitivity in LaAlO_3_:Er^3+^,Yb^3+^ systems [33]. The incorporation of Nd^3+^ and Yb^3+^ sensitizers further enables dual-wavelength excitation, balancing sensitivity and biological compatibility. By strategically isolating emitting centers within an active shell architecture, this design minimizes detrimental CR, resulting in highly linear luminescence intensity ratio (LIR) responses and improved sensitivity under biologically relevant conditions (Fig. 9) [33]. These studies collectively demonstrate that intrinsic sensing performance is governed by the control of nonradiative processes and energy transfer pathways. Shell engineering, dopant distribution, and excitation strategy all contribute to enhancing signal stability and sensitivity. Compared to conventional probes, UCNPs offer the advantage of low background interference and deeper tissue operability, though challenges remain in optimizing brightness under low-power excitation and ensuring reproducibility across different host compositions.

#### 5.1.2 Interfacial biosensing and molecular diagnostics

**Fig. 10:**
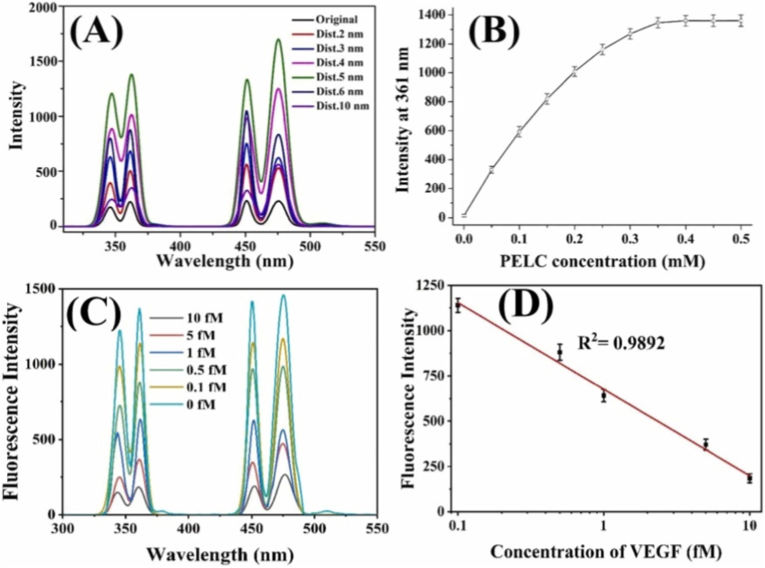
(**a**) Luminescence spectra with varying UCNP-AuNP gaps. (**b**) PELC concentration optimization. (**c**) VEGF-dependent emission changes. (**d**) Calibration curve for VEGF quantification [151]. Copyright 2024. Reproduced with permission from Elsevier.

In contrast to intrinsic sensing, biosensing and analytical diagnostics rely on engineered interfaces that translate molecular recognition events into changes in UCNP emission. Here, UCNPs function as optical reporters within hybrid systems, and performance depends on the efficiency and stability of interactions between UCNPs and external components such as DNA, proteins, dyes, or plasmonic nanostructures. One of the most advanced examples of interfacial design is the plasmon-enhanced luminescence complex (PELC) reported by Khan et al., which integrates NaYF_4_:Yb,Tm UCNPs with glutathione-coated AuNPs through DNA-guided assembly [151]. By precisely controlling interparticle spacing, the system exploits plasmonic coupling to enhance upconversion emission via the Purcell effect. In this context, the localized plasmonic field directly amplifies the radiative decay rate of the Tm^3+^ activator states following ETU excitation. This architecture enables ultrasensitive detection of vascular endothelial growth factor with attomolar sensitivity, demonstrating how nanoscale control over donor-acceptor interactions can dramatically amplify signal output (Fig. 10) [151]. However, such systems also highlight a key limitation: their dependence on precise structural organization may restrict scalability and robustness in real-world applications.

Another direction involves ratiometric and dual-excitation sensing strategies that improve reliability in complex biological environments. Zhou et al. developed dye-sensitized UCNP nanoprobes for ATP detection, where analyte binding disrupts LRET pathways between the UCNP donor and the dye acceptor, producing quantifiable changes in emission under dual NIR excitation [34]. This approach minimizes environmental interference and enables in vivo validation, illustrating how combining optical design with molecular recognition enhances analytical performance. More complex biosensing systems incorporate multifunctional architectures, such as CRISPR-Cas12a-integrated UCNP platforms for nucleic acid detection [11], or smartphone-based diagnostic devices for viral RNA analysis [152]. These systems extend UCNP functionality beyond simple signal transduction toward integrated diagnostic platforms, though they often introduce additional complexity in fabrication and operation. Given the breadth of approaches, a comprehensive comparison of representative UCNP-based sensing systems is provided in Table 3.

**Table 3:** Representative UCNP systems for sensing and analytical diagnostics applications.

Application Class	Representative Study /System	UCNP Design	Sensing Mechanism	Key Performance	Main Advantage	Main Limitation	Reference
Mechanical Force Sensing	Alkaline-Earth Rare-Earth UCNPs (NaYF_4_, CaLuF, SrLuF, BaLuF, SrYb_0.72_Er_0.28_F@SrLuF)	Core-shell UCNPs doped with Yb^3+^ (sensitizer) and Er^3+^ (emitter); host lattices varied (Na, Ca, Sr, Ba)	Ratiometric spectral change (red-to-green emission ratio) under applied pressure in diamond anvil cell	Best sensitivity: 0.26 ± 0.04 GPa/√Hz (SrYb_0_._72_Er_0_._28_F@SrLuF); brightness up to 12,000 cps	Bright emission with high mechano-sensitivity; near-IR excitation allows deep tissue, low phototoxicity	Brightness decreases with smaller particle size; synthesis complexity (multiple shelling, doping control)	[150]
Temperature Sensing (Microfluidics)	3D printed optofluidic chip with embedded UCNP emitters	NaYF_4_:Yb^3+^,Er^3+^ UCNPs doped in UV-curable matrix, integrated into microfluidic channels	Ratiometric photoluminescence (green bands at 521 nm and 541 nm) under 980 nm NIR excitation	Stable, narrow fluorescence enabling accurate local temperature measurement; demonstrated in microalgae culture medium	Nonintrusive, real-time, precise optical temperature sensing; compatibility with biological/biomedical settings	Integration requires photolithography and embedding steps; performance limited to spectral resolution and host stability	[153]
Temperature sensing	Nanocrystalline Y_2_O_3_:Er^3+^,Yb^3+^ UCNPs	Er^3+^ (emitter) and Yb^3+^ (sensitizer) co-doped Y_2_O_3_ nanoparticles	Ratiometric luminescence thermometry (green/red emission ratio under 980 nm excitation)	Demonstrated reliable nanothermometry in vitro; emission stable under biological conditions	Dual functionality: bioimaging and local temperature sensing; biocompatible oxide host	Lower upconversion efficiency compared to fluoride hosts; brightness limited	[154]
Temperature Sensing (Nanothermometry)	LaAlO_3_:Er^3+^,Yb^3+^ nanoparticles with inert or Nd^3+^/Yb^3+^ doped shells	Core@shell UCNPs: Er^3+^,Yb^3+^ doped LaAlO_3_ core; coatings of undoped LaAlO_3_ or Nd^3+^/Yb^3+^ doped shells	Ratiometric luminescence thermometry (green emission bands at 525 nm and 545 nm) under 808 nm and 980 nm excitation	Relative thermal sensitivity up to 1.79% K^−1^ at 305 K (980 nm excitation); excited state lifetimes 56-68 μs	Enhanced green emission intensity (up to 4×); dual excitation channels (808 nm safer for biological media, 980 nm higher sensitivity)	Complex multi-step synthesis; Nd^3+^/Yb^3+^ co-doping reduces sensitivity compared to inert shell	[33]
Photothermal nanoheaters with real-time temperature sensing	Dye-sensitized NaYF_4_:Yb^3+^/Er^3+^@NaYF_4_:Yb^3+^/Nd^3+^ core/shell UCNPs functionalized with IR-806 dye.	Tri-doped core/shell NaYF_4_:Yb^3+^/Er^3+^ (core) with NaYF_4_:^3+^/Nd^3+^ (shell), surface functionalized by IR-806 dye.	Energy transfer cascade: IR-806 dye → Nd^3+^ (shell) → Yb^3+^ (shell/core) → Er^3+^ (core), enabling ∼800 nm excitation upconversion.	Up to 28-fold enhancement in emission intensity; ∼20× stronger UCL compared to Yb-only systems; improved photothermal conversion and temperature sensing under 808 nm excitation.	Shift from 980 nm to biocompatible 800 nm excitation, reducing tissue overheating; strong luminescence and dual functionality (UCL + photothermal).	Performance highly dependent on Nd^3+^ concentration; excessive Nd^3+^ leads to CR and quenching; dye binding stability requires careful optimization.	[155]
Biosensing /biomedical imaging	Photon UCNPs in whole human blood	Core/shell NaErF_4_/NaYF_4_ (compared with NaYF_4_:Yb,Er; NaYF_4_:Yb,Tm; NaTmF_4_/NaYF_4_)	Lanthanide ion upconversion (Er^3+^, Tm^3+^, Yb^3+^ sensitization; GSA/ESA and ET processes)	Emission detectable through 3.0 mm blood layer at 1532 nm; penetration depth up to 7.5 mm	Deep NIR-II/III penetration, reduced autofluorescence, reliable luminescence in whole blood	Lower emission intensity at 975 nm vs Yb/Er systems; quenching <650 nm; blood absorption/scattering reduces signal	[156]
Optical thermometry /biomedical sensing	AgGd(MoO_4_)_2_:Er^3+^/Yb^3+^@mSi core-shell UCNPs	Core-shell nanoplates with cooperative ligand field enhancement; mesoporous silica coating	Ratiometric luminescence thermometry via Er^3+^ green/red emission ratio (^2^H_11_/_2_, ^4^S_3_/_2_ → ^4^I_15_/_2_ vs ^4^F_9_/_2_ → ^4^I_15_/_2_)	High sensitivity (S = 1.23% K^−1^ at 313 K); linear response in physiological range	Enhanced emission via cooperative ligand field; good biocompatibility; silica shell improves dispersion	Requires precise calibration; limited temperature range; potential photobleaching under prolonged excitation	[157]
Optical thermometry /security inks	NaYF_4_:Er^3+^,Yb^3+^@NaYF_4_:Yb^3+^	Core NaYF_4_:Er^3+^,Yb^3+^ with sensitizing shell to enhance energy transfer	Ratiometric luminescence thermometry (green/red Er^3+^ emission ratio) and UCL for ink authentication	Improved emission intensity; higher sensitivity for temperature readout; visible security ink patterns	Shell sensitization boosts energy transfer efficiency; dual-use in biomedical sensing and anti-counterfeiting	Requires careful shell engineering; performance depends on excitation stability; limited to specific host lattice	[158]
Optical thermometry /OCT imaging	PEG-coated NaGdF_4_:Ho^3+^/Yb^3+^ nanoparticles	NaGdF_4_ host lattice doped with Ho^3+^/Yb^3+^, PEG surface coating for biocompatibility	Ratiometric luminescence thermometry (Ho^3+^ emission bands) + OCT contrast enhancement	Bright UC luminescence; improved OCT contrast; reliable temperature readout in physiological range	Dual functionality (imaging + thermometry); PEG coating improves dispersion and biocompatibility	Requires precise calibration; possible signal attenuation in complex tissues; limited to specific excitation wavelengths	[159]
Biosensing (VEGF detection)	PELC for VEGF	NaYF_4_:Yb,Tm UCNPs (∼27 nm, hexagonal phase) combined with glutathione-coated AuNPs (∼4.6 nm) via ssDNA scaffold	Plasmon-enhanced UCL; aptamer substitution for VEGF binding	Enhancement factor ∼4.4 at 361 nm (optimal 5 nm gap); LOD = 25.1 aM; linear range 0.1-10 fM; stable for 1 month at 4°C	Ultrasensitive VEGF detection; high specificity; robust DNA scaffold; compatible with human serum samples	Requires precise gap control; weak Purcell effect for IR emission; limited validation (47 donors)	[151]
Biosensing /bioimaging	Carboxyl-functionalized UCNPs for Exo I detection and in vivo imaging	Two types: NaLuF_4_:Yb/Er (green emission) and NaLuF_4_:Gd/Yb/Tm (NIR/blue emission); both capped with carboxyl groups via malonic acid	DNA-conjugated UCNPs quenched by graphene oxide; Exo I hydrolysis restores luminescence; UCNP luminescence for in vivo imaging	Exo I detection limit: 0.02 U/mL; strong green (540 nm) and NIR (800 nm) emissions; stable imaging in nude mice	One-step synthesis, hydrophilic UCNPs, high carboxyl density for bioconjugation, strong anti-photobleaching	Mixed cubic/hexagonal phases; requires precise GO-DNA interactions; biodistribution mainly in liver	[102]
Biosensing (enzyme monitoring)	Optical fiber probe for tyrosinase detection	β- NaYF_4_:Yb^3+^,Er^3+^ UCNPs (∼120 nm, hexagonal phase), surface modified with polyacrylic acid (PAA) for fiber immobilization	Photo-induced electron transfer (PET) between UCNPs and dopaquinone (DQ) generated from dopamine oxidation catalyzed by tyrosinase	Detection limit: 0.028 U/mL; linear range: 0.1-0.6 U/mL; recovery in human serum: 96-105%; repeatability RSD: 0.3-2.3%	Portable, reusable fiber-based sensor; in situ monitoring; high selectivity against interfering biomolecules	Detection limit higher than solution-phase sensors; requires 60 min incubation; limited penetration depth	[160]
biosensing (viral protein immunoassay)	LiYbF_4_:Tm^3+^@LiYF_4_ UCNPs for singlet oxygen generation and SARS-CoV-2 immunoassay	Core-shell LiYbF_4_:Tm^3+^@LiYF_4_ nanoparticles; high Yb^3+^ content for efficient sensitization	UCL (980 nm excitation → blue emission); energy transfer to photosensitizer for singlet oxygen; UCNP-based immunoassay for viral nucleoprotein	Bright blue emission; efficient singlet oxygen generation; sensitive immunoassay with low LOD (594 pg/mL)	High brightness and efficient sensitization enabling both photochemical activity and sensitive biosensing	Requires high excitation power; limited penetration depth of blue emission; immunoassay validation limited to lab scale	[161]
Biosensing (periodontitis diagnosis)	Disk-like G-UCNPs-LFIS for multiplex detection of MMP-8, IL-1β, TNF-α in GCF	Green core-shell NaYF_4_:Yb,Er@NaYF_4_ UCNPs, PAA-modified for antibody conjugation	Immuno-sandwich assay on lateral flow strip; UCNP luminescence readout	Detection limits: 5.455 ng/mL (MMP-8), 0.054 ng/mL (IL-1β), 4.439 ng/mL (TNF-α); recovery rates 90-110%; correlation with clinical methods: 0.995 (MMP-8), 0.976 (IL-1β), 0.977 (TNF-α); total assay time 30 min	Multiplex detection of three biomarkers in GCF; high sensitivity and specificity; rapid chair-side diagnosis; strong correlation with clinical assays	Requires antibody optimization; variability at very low concentrations; larger sample size needed for cut-off validation	[162]
Biosensing (ATP detection)	Cypate-sensitized UCNP nanoprobes	NaYF_4_:Yb,Er UCNPs conjugated with Cypate dye; ATP aptamer integrated for recognition	Ratiometric luminescence sensing: ATP binding alters energy transfer between UCNPs and Cypate, shifting emission ratio	Detection limit: ∼0.23 nM ATP; linear range: 0.5-500 nM; demonstrated intracellular ATP imaging and in vivo mouse model detection	High sensitivity and selectivity; effective intracellular and in vivo application; ratiometric design reduces background interference	Requires dye-UCNP conjugation stability; potential photobleaching of Cypate; limited validation in complex tissues	[34]
Biosensing (ATP detection)	UCNPs-cDNA-aptamer-Cy3 nanosensor	NaYF_4_:Yb^3+^,Er^3+^ UCNPs (∼30 nm, hexagonal phase), silica-coated and amino-modified for cDNA conjugation	LRET between UCNP donor and Cy3 acceptor; ATP binding dissociates aptamer, restoring UCNP fluorescence	Linear detection range: 1-1000 μM ATP; correlation R^2^ = 0.9922; recovery in serum: 96.3-102.4%; cell viability >80% at 400 μg/mL; successful intracellular ATP imaging in U251MG glioma cells	Rapid response (10 min); high selectivity against ATP analogs (UTP, CTP, GTP); good biocompatibility; applicable in serum and live-cell imaging	Requires high Cy3-aptamer optimization; limited validation in animal imaging; fluorescence recovery dependent on aptamer stability	[163]
Biosensing (multiplex bioassays)	UCNP-encoded microcarriers fabricated via electrospray microfluidics	NaYF_4_:Yb,Er/Tm UCNPs embedded in polymer microcarriers; distinct emission codes for multiplexing	Encoded luminescence signatures allow simultaneous detection of multiple analytes in one assay	High throughput multiplexing; stable UCNP emission; reproducible microcarrier fabrication; demonstrated multiplex bioassay feasibility; LOD = 1.455 ng/ml	Simple, scalable electrospray fabrication; robust UCNP encoding; enables point-of-care multiplex diagnostics	Requires precise code calibration; emission overlap possible; limited clinical validation	[164]
Biosensing (nucleic acid detection)	UCNP-DNA nanohybrids for FRET assays	Core-shell β-NaYF_4_:Yb,Er@NaYF_4_ UCNPs (∼28 nm) capped with thin poly(sodium 4-styrene sulfonate) (PSS) polymer; DNA strands covalently linked via sulfonamide bonds	FRET between UCNP donor (Er^3+^ green emission) and Cy3 dye acceptor upon DNA hybridization; ratiometric readout	DNA hybridization assay (LOD ≈0.3 ± 0.1 nM); miR20a assay (LOD ≈30 ± 10 pM (4.5 fmol)), dynamic range 0.01-10 nM; >20-fold lower LOD than Tb-QD FRET assays	Thin PSS coating preserves UCNP luminescence, ensures DNA accessibility, enables wash-free rapid assays; stable nanohybrids for months	Aggregation in PBS due to phosphate ions; hybridization efficiency depends on DNA density and buffer conditions; optimization needed for broader targets	[36]
Biosensing (pathogen detection)	NaBiF_4_:Yb^3+^,Er^3+^ UCNP-based ECL immunosensor for *E. coli* O157:H7	Hexagonal NaBiF_4_:Yb^3+^,Er^3+^ UCNPs (∼228 nm) synthesized at room temperature; combined with Au NPs and anti-*E. coli* O157:H7 antibody	Electrochemiluminescence with K_2_S_2_O_8_ coreactant; sulfate radicals amplify UCNP ECL; antigen-antibody binding reduces signal	Linear range: 200-100,000 CFU/mL; LOD: 138 CFU/mL; reproducibility RSD: 0.8-2.2%; stability: retains ∼93.5% signal after 2 weeks at 4°C	Fast, room-temperature synthesis of environmentally friendly NaBiF_4_ UCNPs; high sensitivity and specificity; robust repeatability and stability	Larger particle size (∼228 nm) may limit surface area; validation limited to lab samples; requires 45 min incubation	[39]
Biosensing (apoptosis monitoring)	UCNP@PDA@Cy3-pep nanoplatform for caspase-3 detection and therapy evaluation	Core@shell@shell NaErF_4_:Tm^3+^@NaYbF_4_@NaYF_4_:Nd^3+^ UCNPs (∼33 nm), PDA shell (∼8 nm), Cy3-labeled peptide (DEVD substrate + PSP tumor-targeting motif), STS drug loading	FRET between Cy3 donor and PDA acceptor; caspase-3 cleavage of DEVD breaks FRET → Cy3 fluorescence recovery; UCNP red emission (654 nm) as internal reference; PDA provides PTT; PDA also loads staurosporine (STS) for chemotherapy	Caspase-3 detection: linear range 0.5-50 ng/mL; LOD 0.065 ng/mL (UCNP@PDA@Cy3-pep), 0.073 ng/mL (STS-loaded); intracellular detection LOD ≈448 cells; STS loading capacity ≈0.93 mg/mg UCNP@PDA; photothermal conversion efficiency ≈21.8%; tumor accumulation ≈14%ID/g; in vivo synergistic therapy: 4.5-fold higher signal under 1.8 W/cm^2^ laser	Six-in-one functionality: (i) UCNP internal reference, (ii) PDA FRET acceptor, (iii) caspase-3 substrate DEVD, (iv) PSP tumor-targeting motif, (v) PDA photothermal agent, (vi) STS chemotherapy drug; enables real-time, noninvasive monitoring of apoptosis during therapy	Requires laser irradiation (808 nm, 1.5-1.8 W/cm^2^); STS release only partial (∼3% burst under acidic pH); selectivity challenged by caspase-7 cross-reactivity; in vivo validation limited to MG-63 xenograft model	[165]
Biosensing (viral RNA detection)	Smartphone-based PULD (POC UCL diagnostics) for SARS-CoV-2 N gene	Core-shell NaGdF_4_:Yb^3+^/Er^3+^@NaGdF_4_ UCNPs (∼18 nm, hexagonal phase), polyacrylic acid (PAA) modified for oligo conjugation; paired with 4.5 nm Au NPs functionalized with thiolated DNA probes	LRET: hybridization brings UCNPs and Au NPs into proximity → UCNP emission quenched → smartphone-readable signal	LOD: 11.46 fM without target amplification; linear range: 200 fM - 10 nM; turnaround time: 20 min; 100% concordance with RT-PCR in Omicron variant clinical samples	Amplification-free, ultrasensitive detection; portable smartphone-controlled device; rapid workflow (sample-in, answer-out); strong specificity against mismatched sequences	Requires RNA extraction step; sensitivity depends on probe design; tested on limited clinical cohort (9 samples); device throughput not yet optimized	[152]
Biosensing (antibiotic detection)	UCNP@SiO_2_@silk fibroin biosensor for roxithromycin (RXM) and azithromycin (AZM)	NaYF_4_:Yb^3+^,Er^3+^,Gd^3+^ UCNPs (∼26 nm) synthesized in OA/ODE, coated with ∼5.4 nm SiO_2_ shell; silk fibroin corona (∼2.3 nm) hardened by ethanol treatment	FRET + charge-transfer effect: UCNP donor emission (540 nm) quenched by AR-RXM complex acceptor immobilized in silk fibroin corona; synergistic quenching (79% FRET, 21% IFE)	RXM detection in deionized water: range 1.0-141.6 nM, LOD 0.682 nM; RXM detection in river water: LOD 0.98 nM; AZM detection: range 0.99-117.6 nM, LOD 0.77 nM; stability >90% FI retained after 60 days in water	Aptamer-free, low-cost design; silk fibroin provides biocompatible, stable corona; high sensitivity in both lab and environmental samples; versatile for multiple antibiotics	Specificity strongest for RXM; partial cross-response to erythromycin; performance depends on SF:UCNP ratio (aggregation at high SF); validation limited to environmental water, not yet food matrices	[166]
Biosensing (nucleic acid detection)	CRISPR-Cas12a dual-mode UNSC biosensor	Core-shell NaYF_4_:Yb^3+^/Er^3+^@NaYF_4_ UCNPs (∼53 nm) coated with SiO_2_ (∼80 nm) and decorated with ultrasmall CeO_2_ nanodots (∼5 nm); ssDNA probes covalently attached	Dual-mode: (i) UCL recovery after Cas12a cleavage releases UCNPs from magnetic graphene oxide (MGO), (ii) Peroxidase-like activity of CeO_2_ nanozyme catalyzes TMB oxidation for colorimetric readout	LOD: 320 fM (UCL mode), 28.4 pM (colorimetric mode); linear ranges: 0.5 pM-200 pM (UCL), 50 pM-1000 pM (colorimetric); detection time ∼1.5 h; recovery 94-107% (UCL), 85.7-103.7% (colorimetric) in plasma and BALF	Amplification-free detection; dual-mode readout increases reliability; strong specificity against mismatched and non-target viral DNAs; stable in complex matrices (blood plasma, BALF)	Requires Cas12a enzyme and crRNA preparation; colorimetric mode less sensitive; current design limited to single-target detection (S and Orf genes of SARS-CoV-2)	[11]
Nanomaterial engineering (biosensing enabling technology)	Hybrid strategy for ultrasmall UCNPs	NaYF_4_:Yb^3+^,Er^3+^, Gd^3+^ UCNPs (∼12 nm) synthesized via solvothermal method; hybridized with dye molecules and plasmonic nanostructures	Dual sensitization: (i) organic dye antenna broadens absorption, (ii) plasmonic coupling enhances emission; synergistic energy transfer	∼12-fold luminescence enhancement compared to bare UCNPs; stable emission under continuous excitation; retained colloidal stability	Enables ultrasmall UCNPs (<15 nm) with strong luminescence, suitable for bioimaging and biosensing; hybrid approach is versatile	Complexity of hybrid assembly; dye stability under long irradiation; plasmonic coupling requires precise spacing	[167]
Biosensing (protein detection)	Tag-LIBS immunoassay for human serum albumin (HSA)	Streptavidin-modified NaYF_4_:Yb^3+^,Er^3+^ UCNPs used as labels in ULISA format	LIBS readout of yttrium emission (Y II 371.03 nm) from UCNP tags; comparison of single pulse (SP) vs. collinear double pulse (DP) excitation	LOD (immunoassay): SP LIBS 0.51 ng/mL (6.5 μg/mL UCNP), 3.4 ng/mL (3.25 μg/mL UCNP); DP LIBS 0.29 ng/mL (6.5 μg/mL UCNP), 0.67 ng/mL (3.25 μg/mL UCNP). LOD (ULISA luminescence): 14-40 pg/mL depending on UCNP concentration. LOD (ELISA): 0.37 ng/mL. Working range: ∼2-70 ng/mL. Successful analysis of spiked urine samples (0.1-10 ng/mL HSA).	DP LIBS improves sensitivity up to ∼5× compared to SP; LIBS readout approaches ELISA sensitivity; UCNP labels enable elemental imaging and multiplexing potential	DP advantage depends on UCNP concentration (stronger at lower label concentration); matrix effects limit sensitivity; ULISA luminescence still outperforms LIBS	[168]
Biosensing (antioxidant detection)	NaGdF_4_:Yb^3+^,Er^3+^ UCNPs grown in situ inside desilicated ZSM-5 zeolite (DSZSM-5/HT)	NaGdF_4_:Yb,Er UCNPs synthesized inside enlarged mesopores of DSZSM-5; mild heat treatment at 400°C enhances crystallinity and emission	Gallic acid reduces oxidized TMB (oxTMB) → recovery of UCNP red emission; ratiometric change in I_R_/I_G_ used for quantification	LOD: 0.0095 mmol/L (9.5 μM); linear range: 0-0.938 mmol/L; recovery in spiked tea samples: 95-104%; strong anti-interference against ions and biomolecules	In situ growth inside DSZSM-5 yields tightly bound UCNP-zeolite interfaces, drastically enhancing emission (up to 2-3 orders of magnitude); robust selectivity and sensitivity	Requires Ag^+^/TMB chemistry; detection limited to solution phase; performance depends on zeolite pore size (DSZSM-5 superior to DSFAUY)	[37]
Biosensing (miRNA detection)	UCNP-DNA probe for miRNA-222	NaLiErF_4_:Tm core UCNPs (Li^+^ 12%, Tm^3+^ 1.5%) synthesized solvothermally; coated with NaGdF_4_:Yb active shell (∼6-8 nm)	FRET between UCNP red emission (654 nm) and BHQ-2 quencher attached to DNA hairpin; hybridization with target miRNA-222 opens hairpin, separates BHQ-2, restoring UCNP emission	LOD: 0.077 nM; linear range: 0.5-2.5 nM; LOQ: 0.257 nM; recovery in serum: 97.6-102.1%; RSD <5%; UCL intensity enhancement: 6× (Li^+^/Tm^3+^ core), 32× (core-shell vs. bare core)	Strong red emission in biological optical window; amplification-free detection; high specificity for miRNA-222; validated in serum samples with agreement to qRT-PCR	Requires 60 min incubation; selectivity limited to designed DNA hairpin; performance depends on precise Li^+^/Tm^3+^ ratios and shell thickness	[169]
Biosensing (xanthine detection)	UCNP-Try-chy-AuNPs-AuNCs dual-readout probe	CTAB-stabilized NaYF_4_:Yb^3+^,Er^3+^ UCNPs (∼30-40 nm) synthesized hydrothermally	Inner Filter Effect (IFE) quenching of UCNP green emission (520-540 nm) by Try-chy-AuNPs-AuNCs; cascade amplification via XAO-catalyzed XA → H_2_O_2_ → Fe^2+^/I^−^ → I_2_ etching AuNCs, restoring UCNP emission and colorimetric signal	LOD (fluorescence): 26.3 nM for XA; linear ranges: 0.06-5.0 μM and 5.0-80 μM; LOD (fluorescence): 22.6 nM for H_2_O_2_; dual readout (fluorescence + colorimetric); recovery in human serum: 94.6-105%; RSD 2.36-5.65%	Ultrafast (3 min), green synthesis of monometallic AuNPs-AuNCs hybrids; high quenching efficiency (>90%); cascade amplification boosts sensitivity; dual readout increases reliability	Requires enzymatic step (XAO); selectivity limited to purine metabolites; matrix effects possible in complex biological fluids	[170]
Biosensing (antibiotic detection)	Dual-recognition UCNP-based IFE biosensor for sulfadimethoxine (SDM) in aquatic samples	Lanthanide-doped UCNPs (NaYF_4_:Yb^3+^,Er^3+^, ∼20 nm, OA-coated, ligand-exchanged with alendronic acid for water solubility)	Inner Filter Effect (IFE) via overlap of UCNP emission (∼655 nm) with HRP-catalyzed oxTMB absorption; dual recognition by aldehyde-functionalized MNPs and SDM-specific aptamer	Linear range: 0.5-1000 ng/mL; LOD: 0.13 ng/mL; R^2^ = 0.9912; recoveries in spiked aquatic samples: 88.41-96.78% with RSD 1.40-9.20%	Dual-role recognition (MNPs + aptamer) enhances selectivity and sensitivity; stable UCNP fluorescence under varying pH and temperature; validated against HPLC with no significant difference	Recognition cross-reactivity with other sulfonamides possible; requires aptamer and MNP functionalization steps; matrix effects may complicate broader food applications	[171]
Glucose Monitoring	UCNP@Ag-GOx nanoprobe for glucose detection and cancer cell discrimination	NaGdF_4_:Yb^3+^,Er^3+^ UCNP core (∼21 nm) coated with thin Ag shell (∼25 nm total size); surface anchored with glucose oxidase (GOx) via glutaraldehyde	Fluorescence turn-on via Ag shell quenching/recovery: glucose → GOx → H_2_O_2_ generation → Ag shell etching → restored UCNP emission	Linear range: 0-3.2 μM glucose; LOD: 1.77 μM; R^2^ = 0.9953; 5-fold emission intensity difference between HeLa (cancer) and MRC-5 (normal) cells; stable in PBS and H_2_O for 7 days	Ultrasensitive detection with ultralow LOD; strong selectivity against proteins, amino acids, saccharides, and ions; enables in vitro optical bioimaging to distinguish cancer vs. normal cells	Limited dynamic range (μM scale only); requires Ag shell stability and GOx immobilization; not yet validated in vivo or in complex serum matrices	[172]
Antibiotic Detection	Competitive assay using surface-modified UCNPs for ofloxacin	β-NaYF_4_:Yb^3+^,Er^3+^ hexagonal UCNPs (∼52 nm) synthesized solvothermally; silica-coated (∼10 nm shell) and functionalized with mal-PEG-silane for thiol-reactive groups; conjugated with reduced anti-ofloxacin IgG half-antibodies	Competitive immunoassay: UCNP-antibody conjugates compete with plate-bound ofloxacin-BSA conjugates; detection via UCNP photoluminescence (green emission ∼541 nm)	Detection limit: ∼10 nM; linear range: 0.1 mM-10 nM; R^2^ = 0.9904; high signal-to-noise ratio; selective against sulfamethazine (no cross-reactivity)	Rapid, single-step competitive assay; high sensitivity compared to ELISA; stable UCNP fluorescence; adaptable to other biomarkers	Silica coating introduces partial quenching (OH groups); requires antibody cleavage and conjugation steps; detection limit still higher than aptamer/affimer-based approaches	[173]
Hydrogen Peroxide Detection	Core-satellite UCNC@MOF/NiS_x_ composites for H_2_O_2_ sensing	β-NaYF_4_:Yb,Er (33.2 nm, PVP-modified) combined with ZIF-8 (∼720 nm) or UiO-66-NH_2_ (∼200 nm); also β-NaYF_4_:Yb,Tm UCNCs for ratiometric detection	NiS_x_ nanoparticles quench UCNP emission; H_2_O_2_ decomposes NiS_2_, restoring UCNP luminescence (I/I_0_ for Er^3+^, or I_476_/I_802_ for Tm^3+^)	LOD: 0.76 μM (NaYF_4_:Yb,Er@ZIF-8/NiS_x_); 0.38 μM (NaYF_4_:Yb,Er@UiO-66-NH_2_/NiS_x_); 0.15 μM (NaYF_4_:Yb,Tm@UiO-66-NH_2_/NiS_x_, ratiometric)	Tunable emission via ion doping; flexible composite design; high surface area MOFs improve adsorption and stability; strong selectivity against ions, amino acids, saccharides	Requires NiS_x_ loading and precise composite assembly; quenching efficiency depends on overlap; not yet validated in biological fluids	[174]
Hormone detection	Ratiometric upconversion-luminescence in-situ sampling aptasensing platform integrated with smartphone device for 17β-estradiol	Core/inner-shell/outer-shell NaYF_4_@NaYbF_4_:Tm@NaYF_4_ UCNPs (∼34.4 nm, PAA-modified, aptamer/dsDNA functionalized, SYBR Green I acceptor)	LRET: UCNP donor → SYBR Green I acceptor; target 17β-E2 binding disrupts LRET, restoring UCNP emission (ratiometric I_477_/I_646_)	Linear range: 0.02-200 ng/mL (spectra), 0.05-200 ng/mL (smartphone imaging); LOD: 0.015 ng/mL (spectra), 0.046 ng/mL (smartphone); recoveries in real samples: 80.42-91.83% (spectra), 83.72-96.75% (imaging); CVs 1.24-8.55%	Dual-mode readout (spectrometer + smartphone); in-situ sampling with μPADs and PMMA needle; strong self-calibration via three detection zones; portable and low-cost	Slightly lower recoveries vs. ELISA due to chromatography/sample loss; not yet validated for direct human in-situ screening; limited to single-target detection	[175]
Multiplex endocrine sensing	Ratiometric aptasensor for simultaneous detection of bisphenol A (BPA) and 17β-estradiol (E2)	Three core-shell UCNPs: NaYF_4_:Yb/Tm@NaYF_4_ (blue, 474 nm), NaYF_4_:Yb/Er@NaYF_4_ (green, 542 nm), NaHoF_4_@NaGdF_4_/Yb (red, 658 nm reference); PAA-modified, aptamer-conjugated	LRET between UCNP donors and metallic MoS_2_ nanosheets; aptamer-target binding disrupts quenching, restoring UCNP emission; red UCNPs serve as internal reference	Separate detection: BPA linear 0.01-20 ng/mL, LOD 0.01 ng/mL; E2 linear 0.05-20 ng/mL, LOD 0.05 ng/mL. Simultaneous ratiometric detection: BPA linear 0.1-20 ng/mL, LOD 0.04 ng/mL; E2 linear 0.2-20 ng/mL, LOD 0.1 ng/mL. Recoveries in tap water/milk: 90.7-112.9%	Simultaneous detection of two estrogens; ratiometric design improves accuracy and reliability; strong selectivity against other estrogens/phenols; validated in real food samples	Requires aptamer conjugation and MoS_2_ optimization; sensitivity slightly reduced in dual-target mode compared to separate detection; limited to BPA and E2	[176]

#### 5.1.3 Comparative performance and sensing strategy trade-offs

Across sensing applications, a clear distinction emerges between systems driven by intrinsic photophysical responses and those dependent on interfacial engineering. Intrinsic sensing platforms benefit from structural simplicity and high stability, as their performance is encoded directly within the tightly protected UCNP lattice. However, their scope is strictly limited to physical parameters (e.g., temperature and pressure). In contrast, interfacial biosensors offer expansive applicability but introduce severe vulnerabilities at the bio-nano interface. As outlined in Table 3, achieving attomolar or femtomolar detection limits (such as in the PELC or CRISPR-Cas12a systems) requires exceedingly complex architectures, often relying on perfectly tuned Förster resonance energy transfer (FRET) or LRET distances, precisely engineered plasmonic gaps, or multi-step enzymatic cleavages. These stringent structural dependencies restrict scalability, severely limit batch-to-batch reproducibility, and make the sensors highly susceptible to false signals triggered by protein corona formation in whole blood or serum. Future progress in analytical diagnostics must pivot away from merely achieving record-low limits of detection in ideal buffers, and focus instead on simplifying donor-acceptor architectures to ensure signal robustness in highly heterogeneous biological matrices.

### 5.2 Bioimaging and image-guided analysis

The application of UCNPs in bioimaging is fundamentally governed by their ability to generate stable, high-contrast optical signals under NIR excitation while maintaining compatibility with biological environments. Unlike sensing systems, where signal modulation is the primary objective, imaging platforms prioritize photon output, spectral positioning within biological transparency windows, and controlled interactions with physiological media. As a result, UCNP design for imaging is shaped by a balance between optical brightness, colloidal stability, and multimodal integration. Two complementary imaging regimes can be distinguished: conventional imaging, which relies on maximizing emission intensity, and super-resolution imaging, which exploits non-linear excitation behavior and long-lived emission kinetics.

#### 5.2.1 Conventional and multimodal bioimaging

In conventional bioimaging, UCNP performance is primarily determined by emission efficiency under biologically acceptable excitation conditions and the ability to maintain stable dispersion in physiological environments. Core-shell engineering and surface functionalization are therefore central design strategies to protect the upconversion process from environmental quenching. A representative example is the PEGylated NaYF_4_:Yb^3+^/Er^3+^ system developed by Wang et al., which demonstrates how surface modification directly influences biological performance [177]. The strong green emission observed in this system relies on classic ETU pathways under 980 nm excitation. The incorporation of hydrophilic PEG ligands enabled water dispersibility and prolonged circulation, proving that preserving the ETU mechanism in vivo requires robust physical shielding at the bio-nano interface [177].

Beyond passive imaging, targeted and multimodal systems introduce additional layers of functionality. For instance, Li et al. engineered NaErF_4_:Yb@NaGdF_4_:Yb core-shell UCNPs functionalized with tumor-targeting peptides, enabling dual-mode fluorescence and MRI [178]. Here, the active NaGdF_4_:Yb shell not only provides *T*_1_-weighted MRI contrast but actively participates in the energy transfer dynamics, shielding the NaErF_4_ core from non-radiative surface quenching to preserve bright red emission. Similarly, Yang et al. developed multi-shell UCNP architectures integrating fluorescence, computed tomography, and MRI capabilities, highlighting the potential of hierarchical structures to protect internal emitting centers while presenting multiple diagnostic modalities to the biological environment [38]. Additional advances, including oleogel-mediated delivery systems [38], hollow-structured UCNPs for cellular imaging [179], and rapid microwave-assisted synthesis routes [10], further demonstrate the diversity of approaches used to enhance imaging performance. A comparative overview of these strategies, including their design features, imaging modalities, and biological outcomes, is summarized in Table 4.

**Table 4:** Representative UCNP systems for bioimaging applications.

Imaging Class	Representative Study /System	UCNP Design	Imaging Modality	Biological Model /Context	Key Performance	Main Advantage	Main Limitation	Reference
Fluorescence Imaging	Monoclinic Gd_2_O_3_:Yb^3+^/Tm^3+^ UCNPs prepared by laser ablation in liquid	Gd_2_O_3_ host, co-doped with Yb^3+^ (sensitizer) and Tm^3+^ (activator); ∼7.18 nm nanoparticles	Confocal fluorescence microscopy under 980 nm excitation	Murine macrophage cell line (Raw264.7); cytotoxicity tested on CNE-2 and NP69 cells	Strong NIR emission (∼801 nm) more intense than visible bands; visible blue emission (∼481 nm) sufficient for live cell imaging	Excitation and emission both within biological “optical window” → deep tissue penetration, low autofluorescence, reduced photodamage; chemical purity due to laser ablation (no precursors)	Lower upconversion efficiency compared to fluoride hosts; monoclinic phase harder to obtain; visible emission weaker than NIR (limiting naked-eye applications)	[180]
Trimodal Imaging	Multi-shell NaErF_4_:0.5%Tm^3+^@NaYF_4_:20%Yb^3+^@NaLuF_4_@NaGdF_4_ UCNPs	Core@Active-shell@Inert-shell@Gd-shell design; Er^3+^ core, Yb^3+^ sensitizer, Lu^3+^ inert shell, Gd^3+^ MRI shell	Fluorescence (UCL), CT, MRI	In vitro characterization; hydrophilic ligand exchange for water dispersibility (no explicit in vivo biological model reported)	Bright red emission with R/G ratio up to 29; lifetime enhancement (495 μs vs. 55.7 μs); CT HU increase; MRI relaxivity r_1_ = 1.94 mM^−1^ s^−1^	Strong red-dominant emission suitable for bioimaging; trimodal capability (UCL/CT/MRI); water dispersibility via citric acid capping	UCL intensity still lower than traditional NaYF_4_:Yb/Er UCNPs; biological validation limited	[22]
Dual-mode Imaging	PEG-coated NaGdF_4_:Ho^3+^/Yb^3+^ UCNPs	NaGdF_4_ host lattice doped with Ho^3+^ (emitter) and Yb^3+^ (sensitizer); PEG surface coating for biocompatibility	Optical Coherence Tomography (OCT) + Optical Temperature Sensing	In vitro phantom imaging; biocompatibility assessed	Bright red upconversion emission enabling OCT contrast enhancement; temperature sensitivity via emission intensity ratio	Dual functionality (imaging + thermometry); PEG coating improves dispersibility and biocompatibility	Limited biological validation; performance compared mainly in phantom systems; efficiency lower than optimized NaYF_4_ UCNPs	[159]
Fluorescence Imaging	Acetate-modified NaYF_4_:Yb^3+^/Er^3+^ UCNPs synthesized via single-mode focused microwave method	Cubic-phase NaYF_4_ host doped with Yb^3+^ (20%) and Er^3+^ (2%); acetate surface modification for hydrophilicity	Two-photon confocal fluorescence microscopy (980 nm excitation, strong red emission at 669 nm)	HeLa cells (in vitro imaging); cytotoxicity tested up to 128 μg/mL	Strong red emission dominating over green; rapid synthesis (10 min at 150°C); average particle size ∼50 nm; cell viability >75% at high concentration	Fast, energy-efficient synthesis; red emission in biological optical window (650-900 nm) → deeper penetration, reduced autofluorescence; good aqueous dispersibility	Limited to in vitro demonstration; concentration quenching at higher dopant levels; penetration depth still less than NIR-emitting UCNPs	[10]
Fluorescence Imaging	NaErF_4_@NaYF_4_ core-shell UCNPs with Tm^3+^/Ho^3+^ energy capture centers	Core-shell NaErF_4_@NaYF_4_; Er^3+^ emitter, Yb^3+^ sensitizer, Tm^3+^/Ho^3+^ dopants for energy capture; water-dispersible	Upconversion fluorescence imaging under multi-wavelength NIR excitation (980 nm, 808 nm, 730 nm)	In vitro demonstration; aqueous dispersibility emphasized	Tunable emission modulation: red/green/blue balance controlled by excitation wavelength; strong red emission under 980 nm; stable dispersibility in water	Multi-wavelength excitation enables controllable emission color; water dispersibility enhances biocompatibility; potential for multiplexed imaging	Biological validation limited (no detailed in vivo imaging); emission intensity lower than optimized NaYF_4_:Yb/Er systems; complexity of multi-dopant design	[181]
Fluorescence Imaging	OM-coated NaYF_4_:Yb^3+^/Er^3+^ UCNPs incorporated into soybean oil-stearic acid oleogel (UG4 formulation)	β-NaYF_4_ host lattice doped with Yb^3+^ (20%) and Er^3+^ (2%); OM surface coating; dispersed in oleogel matrix	Photoluminescence imaging under 980 nm NIR excitation	In vitro agarose gel diffusion; chicken skin permeation; pig ear skin permeation (human-relevant model)	UG4 showed strongest green emission (∼540 nm); UCNP permeated intact pig ear skin within 48 h; imaging of inner skin layers achieved	First demonstration of transdermal UCNP delivery via oleogel; enhanced permeation due to stearic acid and soybean oil fatty acids; strong PL for optical tracking	Emission dominated by green (autofluorescence interference risk); limited quantitative penetration (μg/cm^2^ scale); majority of UCNP retained in skin layer	[38]
Molecular Imaging	UCNPs incorporated for PTOCT contrast	NaYF_4_:Yb^3+^/Er^3+^ UCNPs	Photothermal Optical Coherence Tomography (PTOCT)	Ex-vivo tissue samples (molecular imaging context)	Enhanced OCT contrast via photothermal effect; UCNPs provide stable signal under NIR excitation	Combines molecular specificity of UCNPs with high-resolution OCT; ex-vivo demonstration shows feasibility	Limited to ex-vivo validation; in vivo translation not yet demonstrated; photothermal heating may pose safety concerns	[24]
Dual-mode Imaging	Peptide-functionalized NaErF_4_:Yb@NaGdF_4_:Yb UCNPs with silica shell	Hexagonal β-NaErF_4_:Yb core (Er:Yb = 85:15) @ NaGdF_4_:Yb shell; carboxyl-terminated silica coating; conjugated with L-SP5-C peptide	UCL (red emission at 655 nm) + T_1_-weighted MRI	In vitro: HCT116, SW620, SW480, NCM460 cells; In vivo: BALB/c nude mice bearing HCT116 xenograft tumors (including ultrasmall ∼13 mm^3^ tumors)	UCL intensity at 655 nm enhanced 12.4-fold vs. core-only; r_1_ relaxivity up to 8.40 mM^−1^ s^−1^ (higher than Gd-DTPA); sensitive detection of ultrasmall tumors	Strong red emission in biological window; peptide (L-SP5-C) provides high tumor affinity; dual-mode imaging enables sensitive early CRC detection	SiO_2_ shell slightly quenches UCL intensity; biodistribution shows liver accumulation; clearance relatively slow; peptide-dependent targeting limited to HCT116 subtype	[178]
Theranostic Imaging	NRhD-PEG-4 nanoparticles (self-assembled organic nanotheranostics)	Rhodamine-derived upconversion dye (NRhD) conjugated with PEG (MW 5000) → self-assembled NPs; pKa = 6.70	UCL (760 nm emission under 808 nm excitation) + PTT (730 nm laser)	In vitro: A549, HeLa, MCF-7, 4T1 cells; In vivo: BALB/c nude mice bearing A549 xenograft tumors	Strong pH-activated UCL “turn-on” in acidic TME; photothermal conversion efficiency up to 18.8% at pH 6.5; tumor ablation with ∼60°C heating under NIR	Acidic TME-specific activation → high tumor selectivity; PEGylation improves solubility, stability, circulation; dual imaging + therapy	Organic dye-based UCNPs less stable than inorganic fluoride hosts; fluorescence weaker in neutral pH; clearance/metabolism relatively fast	[182]
Cellular imaging	Hollow Er^3+^,Yb^3+^ co-doped Y_2_SiO_5_ spheres	Y(OH)CO_3_ core → silica-coated → annealed to monoclinic X1- Y_2_SiO_5_; hollow morphology with nanovoids; doped with Er^3+^ (0.5 mol%) and Yb^3+^ (1.5 mol%)	UCL (green 525-545 nm, red 650 nm) under 980 nm excitation; confocal microscopy at 488 nm	In vitro: U87MG and KNS42 glioblastoma cell lines; cytotoxicity and subcellular localization assays	Strong green and red UCL; perinuclear cytoplasmic localization; >70% cell viability up to 2.0 mg/mL; colloidal stability confirmed (negative ζ-potential)	Hollow morphology enhances luminescence and offers potential for drug loading; low cytotoxicity in GBM cells; stable colloidal dispersions	Emission weaker than fluoride UCNPs; study limited to in vitro glioblastoma cells; cytotoxicity increases at higher concentrations (>2.5 mg/mL)	[179]
Super-resolution microscopy (STED and uSEE nanoscopy)	Controlling the non-linear emission of UCNPs to enhance super-resolution imaging performance	Hexagonal NaYb_x_Tm_1-x_F_4_ nanoparticles with varying Yb/Tm ratios (80/20 to 92/8), with and without thin NaYF_4_ inert shell coating	Stimulated Excitation-Depletion (STED) microscopy and upconversion Super-linear Excitation-Emission (uSEE) microscopy	Single-particle imaging and multiplexed imaging of UCNP mixtures; biological relevance noted but no explicit in vivo model described	Achieved resolutions down to ∼57 nm (STED) and ∼200 nm (uSEE) at reduced excitation powers; slope values up to 7.2 for super-linear emission	Lower excitation/depletion powers reduce photodamage; flexible UCNP composition enables multiplexed super-resolution imaging	Moderate luminescence brightness and long acquisition times (60-180 s), slower than conventional fluorescence super-resolution methods	[98]
Super-resolution multiplexed imaging	Multiplexed structured illumination super-resolution imaging with lifetime-engineered UCNPs	Nd-Yb-Er core-multi-shell τ^2^-dots (active core@energy migration shell@sensitization shell@inert shell)	Time-resolved structured illumination microscopy (TR-SIM)	In vitro nanoparticle imaging	Lateral resolution ∼185 nm; decoding accuracy >93% (3-channel), ∼60-86% (7-channel)	High multiplexing capacity with tailored lifetimes; bright, uniform, photostable probes; single-laser excitation	Reduced accuracy when lifetime curves overlap; limited by detection efficiency and aggregation artifacts	[183]
In vivo luminescence imaging	Direct large-scale synthesis of water-soluble and biocompatible UCNPs	NaYF_4_:Yb^3+^/Er^3+^ (20/2 mol%) nanoparticles, PEG-coated for colloidal stability and circulation	NIR-excited UCL imaging (980 nm excitation, green/red emission)	BALB/c mice (tail vein injection, systemic distribution, liver signal emphasized)	Stable emission under long-term irradiation; colloidal stability >9 months; complete clearance within 72 h; high cell viability up to 500 mg/L	Simple one-pot scalable synthesis (up to 40 g), water solubility, biocompatibility, prolonged circulation, deep tissue penetration	Limited multiplexing capability; emission dominated by Er^3+^ transitions; imaging depth constrained by scattering	[177]
Scaffold-based bioimaging	Natural and synthetic polymer scaffolds impregnated with UCNPs for tissue engineering	Core/shell β-NaYF_4_:Yb^3+^,Er^3+^ /NaYF_4_ nanoparticles (20% Yb, 2% Er, inert NaYF_4_ shell)	NIR-excited UCL (976 nm excitation, green/red emission)	In vitro fibroblast culture (Bj-5ta cells) on collagen, PLGA, and HAGM scaffolds	Stable photoluminescence under 976 nm; R/G ratio shifts with scaffold type; UCNP release detectable; cytocompatibility confirmed with cell proliferation and colonization	Enables real-time scaffold visualization and monitoring of degradation; biocompatible “smart scaffold” platform; adaptable to multiple polymers and fabrication methods	Luminescence quenching and reduced intensity inside scaffolds; emission spectra altered by polymer matrix; no in vivo demonstration yet	[184]

#### 5.2.2 Super-resolution imaging

While conventional UCNP-based imaging focuses on improving penetration depth and signal contrast, recent developments have extended their capabilities toward imaging beyond the diffraction limit. These advances exploit the unique photophysics (nonlinear excitation processes and long emission lifetimes) of lanthanide-doped systems, specifically leveraging the power-dependent saturation of intermediate states found in ESA and high-order non-linear ETU processes. This non-linear emission behavior enables compatibility with super-resolution techniques such as structured illumination microscopy (SIM), stimulated emission depletion (STED), and super-linear excitation-emission (uSEE) imaging. A notable example is the lifetime-engineered UCNP platform developed by Liu et al., where core-multishell architectures are designed to precisely control emission rise and decay kinetics [183]. These distinct temporal signatures, known as τ_2_-dots, are achieved by finely tuning EMU pathways. By deliberately varying the spatial separation and concentration of sensitizer and migrator ions across multiple shells, researchers can orchestrate the energy flow to produce specific lifetimes. These signatures can be selectively resolved using time-resolved SIM, enabling the separation of closely spaced emitters and achieving a lateral resolution of approximately 185 nm [183], which significantly improves imaging precision compared to conventional widefield techniques. Importantly, this approach demonstrates that temporal control over emission can be used as an additional dimension for image reconstruction, expanding the functional space of UCNP-based imaging.

Complementary advances have been reported by Camillis et al., who explored the highly non-linear emission behavior of NaYbF_4_:Tm^3+^ UCNPs to optimize their performance in STED and uSEE microscopy [98]. This super-linear behavior is fundamentally rooted in the multiphoton nature of Tm^3+^ excitation, where high-order ETU and CR dynamics can be optically manipulated by a depletion laser. By tuning dopant ratios and incorporating inert shells to minimize surface defects, the authors achieved steep super-linear emission responses and reduced excitation thresholds, enabling STED resolution down to 57 nm with lower phototoxicity [98]. In addition, the ability to differentiate nanoparticle populations based on their distinct excitation thresholds introduces new opportunities for multiplexed super-resolution imaging within the same spatial domain [98]. Despite these advances, super-resolution UCNP systems remain constrained by several factors, including limited photon budgets, long acquisition times, and the need for precise control over excitation conditions. Furthermore, the complexity of lifetime engineering and nonlinear emission optimization may limit reproducibility across different synthesis batches, posing challenges for widespread adoption.

#### 5.2.3 Comparative imaging performance and resolution constraints

Across bioimaging applications (summarized in Table 4), UCNP systems exhibit a clear progression from brightness-driven conventional imaging toward highly sophisticated architectures capable of multimodal and super-resolution functionality. However, this progression introduces a fundamental optical-biological trade-off. Conventional systems benefit from relatively straightforward ETU dynamics, yielding the high overall photon output necessary for deep-tissue, real-time in vivo tracking, but they remain strictly diffraction-limited. In contrast, advanced super-resolution platforms successfully break the diffraction limit by exploiting the steep non-linear power dependence of ESA and multi-photon ETU. Yet, these highly engineered core-multishell structures often suffer from drastically reduced overall quantum yields. They require prolonged acquisition times (and consequently higher cumulative photon budgets) to gather sufficient signal, making them highly susceptible to motion blur in living systems. Furthermore, translating these complex temporal and non-linear optical dynamics from static in vitro environments to dynamic, highly scattering in vivo tissues remains a formidable challenge. Future developments in UCNP imaging must focus on maximizing the absorption cross-section of these nonlinear probes so that super-resolution and lifetime-multiplexed imaging can be achieved at scanning speeds and irradiance levels compatible with live-tissue survival.

### 5.3 Therapy and theranostics

The therapeutic application of UCNPs is fundamentally rooted in their ability to act as nanoscale transducers that convert deeply penetrating NIR excitation into localized high-energy emission capable of triggering chemical or biological processes. Unlike imaging applications, where photon output is the primary objective, therapeutic systems require efficient energy conversion, controlled interaction with biological environments, and the ability to couple optical activation with functional outputs such as reactive oxygen species generation, heat production, or controlled drug release. As a result, UCNP design for therapy is governed by the interplay between emission engineering, surface architecture, and biological responsiveness.

Two major paradigms dominate this field. Phototherapy-based systems exploit UCNP emission to activate photosensitizers or photothermal agents, enabling spatially controlled cancer treatment. In parallel, UCNP-mediated delivery systems utilize light-triggered or environment-responsive mechanisms to achieve controlled release of therapeutic payloads. Increasingly, these approaches are integrated into multifunctional theranostic platforms that combine imaging, therapy, and monitoring within a single construct.

#### 5.3.1 Phototherapy and cancer theranostics

Phototherapy represents one of the most advanced and clinically relevant applications of UCNPs, where precisely tuned emission enables the activation of photosensitizers for PDT and photothermal agents for PTT. The effectiveness of these systems depends critically on matching UCNP emission profiles with the absorption characteristics of therapeutic agents via efficient energy transfer to maximize tissue penetration while minimizing overheating. A representative example is the NIR-II-responsive UCNP platform developed by Zhao et al., based on a NaErF_4_@NaYbF_4_@NaYF_4_ core-shell-shell architecture [185]. In this system, Er^3+^ ions serve as both sensitizers and activators, enabling emission under 1550 nm excitation, a wavelength within the NIR-II biological window that offers deeper tissue penetration and reduced optical scattering. Because the system utilizes a fully Er^3+^-doped core (NaErF_4_), it is inherently susceptible to severe concentration quenching. To overcome this, the complex shell architecture is designed to manage the spatial distribution of excitation energy, strictly minimizing competitive CR. This precise structural regulation preserves highly efficient ETU pathways among the Er^3+^ ions, enabling the simultaneous generation of both red and green emissions. Upon 1550 nm irradiation, the green ETU emission excites merocyanine 540 (MC540) to generate reactive oxygen species (ROS) for PDT, while the red ETU emission activates iron phthalocyanine (FePc) for localized heating [185]. This dual-mode activation minimizes non-specific tissue overheating and enhances therapeutic penetration.

Beyond dual-mode phototherapy, more complex theranostic systems integrate biological modulation. Chen et al. developed a programmable UCNP-based platform combining PDT with optogenetic immune activation [186]. In this system, dual-wavelength excitation enables sequential activation of different biological processes: blue emission triggers TNF-α release from engineered bacteria, and red emission activates photosensitizers. This sequential activation relies on selectively driving distinct multiphoton pathways within the lanthanide host, illustrating how UCNPs can function as active, programmable regulators of biological responses. Other strategies focus on enhancing therapeutic efficiency through microenvironment modulation or programmable activation, such as incorporating MnO_2_ into UCNP systems to alleviate tumor hypoxia and improve ROS yields during PDT [187], or designing emission-switchable UCNPs for the sequential activation of multiple agents [188]. These approaches demonstrate that the therapeutic performance of UCNPs is not determined solely by optical properties, but also by their ability to interact dynamically with the tumor microenvironment. A broader range of phototherapeutic and theranostic UCNP systems, including polydopamine-based photothermal composites [189], targeted imaging-guided therapy platforms [190], and multimodal chemo-photothermal systems [191], are summarized in Table 5, which compares their design strategies, activation mechanisms, and therapeutic outcomes.

**Table 5:** Representative UCNP systems for therapy and theranostics applications.

Therapeutic Class	Representative Study /System	UCNP Design	Activation Mechanism	Therapeutic Modality	Biological Model	Key Outcome	Main Advantage	Main Limitation	Reference
PDT for malignant breast cancer	UCNPs-NH_2_/PEG-AntiP-Ce6/PT-Her2 nanocomplex	NaYF_4_:Yb,Er UCNPs modified with PEG and antifouling polypeptide, loaded with Ce6, decorated with HER2-targeting peptide	980 nm NIR irradiation excites UCNPs, converting to visible emission that activates Ce6 to generate ROS	ROS-mediated PDT combined with fluorescence imaging	In vitro SKBR-3 breast cancer cells and in vivo 4T1 mouse breast tumor model	Significant ROS generation, apoptosis induction, and complete tumor growth inhibition in vivo	Antifouling performance, high biocompatibility, HER2-targeted selectivity, deep tissue penetration	HER2 targeting less effective in low-expressing tumors; long-term biosafety not fully established	[190]
Cancer therapy (multimodal phototherapy and chemotherapy)	UCNPs@mSiO_2_@MPN-MC540/DOX nanocomposite system	NaYF_4_:Yb,Er,Nd@NaYF_4_:Nd core-shell UCNPs coated with mesoporous silica and Fe-TA metal phenolic network	808 nm NIR laser excitation triggers UCNP upconversion, activating MC540 for PDT and MPN for PTT; acidic tumor microenvironment degrades MPN for DOX release	PDT (MC540), PTT (Fe-TA MPN), and chemotherapy (DOX)	In vitro HeLa cells and in vivo BALB/c nude mice xenograft tumor model	Synergistic PDT/PTT/chemotherapy significantly inhibited tumor growth with minimal systemic toxicity	Multimodal therapy with pH-responsive controlled drug release and imaging guidance	Potential reduced PDT efficacy in hypoxic tumor environments; clearance and long-term toxicity not fully assessed	[191]
Optogenetic therapy (immune + photodynamic combination)	EcN@EL222 + DDUCNP@mSiO_2_-NH_2_-ZnPc	Four-layer dual-emission NaErF_4_:Tm@NaYF_4_@NaYbF_4_:Tm@NaYF_4_ with mesoporous silica coating	980 nm excitation → blue emission activates engineered bacteria (TNF-α release); 808 nm excitation → red emission activates ZnPc photosensitizer (ROS generation)	Immune therapy via TNF-α secretion + PDT via ROS	HeLa cells in vitro; BALB/c nude mice bearing HeLa tumors in vivo	Combination therapy achieved ∼99% tumor cell death in vitro and significant tumor inhibition in vivo	Dual-wavelength NIR activation enables deep tissue penetration and self-clearance of engineered bacteria	Complexity of engineered bacteria system and potential biosafety concerns	[186]
Dual-responsive drug delivery system for anticancer therapy	UCNP@PNBMA/MAA nanocapsules loaded with doxorubicin	NaYF_4_:Yb/Tm UCNP core coated with silica and functionalized with poly(o-nitrobenzyl methacrylate) shell.	Dual-responsive: NIR-triggered upconversion (980 nm) and acidic pH (pH 4.5)	Controlled release of doxorubicin (DOX)	In vitro release studies	Cumulative DOX release reached 59.5% under combined NIR and acidic conditions; loading efficiency 7.23 wt%	Stable nanocapsule structure with dual stimuli responsiveness enabling precise drug release	No in vivo validation; only release kinetics demonstrated in vitro	[192]
PDT with alkaloid berbamine as enhancer	UCNPs@mSiO_2_@Azo@ZnPc&BBM (PB@UA) nanosystem	Four-layer NaErF_4_:Tm@NaYF_4_@NaYbF_4_:Tm@NaYF_4_ core-shell UCNPs coated with mesoporous silica and azobenzene	Sequential NIR excitation: 980 nm triggers BBM release via azobenzene conformational change; 808 nm activates ZnPc photosensitizer	Synergistic PDT enhanced by BBM-mediated suppression of antioxidant defenses and Ca^2+^ dysregulation	HeLa cells in vitro and BALB/c nude mice xenograft tumors in vivo	Tumor inhibition rate of 80.91% in vivo, significantly higher than PDT alone (31.78%) or BBM alone (11.29%)	Programmable dual activation allows precise control of drug release and PDT, overcoming tumor antioxidant defenses	Requires sequential dual NIR irradiation and timing optimization; complexity may limit clinical translation	[188]
Photoacid-triggered drug delivery	DOX-UCSM-CD (doxorubicin-loaded mesoporous silica coated UCNPs with photoacid assistance)	NaYF_4_:TmYb@NaYF_4_ core coated with mesoporous silica (UCS)	NIR (980 nm) irradiation converted to Vis/UV light by UCNPs, triggering merocyanine (MC) photoacid ring-closing reaction and proton release	Controlled drug release (DOX) via acid-labile cap cleavage	HeLa cells (in vitro cytotoxicity assays)	Enhanced DOX release rate and amount; improved anticancer efficacy (65% cell death vs 46% without NIR)	Remote, spatiotemporal control of drug release using NIR-triggered pH manipulation	Demonstrated only in vitro; no in vivo validation or systemic biodistribution data	[193]
Targeted therapy and bioimaging	Tm^3+^/Ho^3+^ doped NaErF_4_@NaYF_4_ UCNPs	Core-shell NaErF_4_@NaYF_4_ nanoparticles, doped with Tm^3+^/Ho^3+^, surface-modified with PAA	Multi-wavelength NIR excitation (808/980/1550 nm)	Bright red luminescence for imaging and potential therapy	No explicit in vivo biological model; water-dispersibility demonstrated	Enhanced red emission intensity, highest R/G ratio with Tm^3+^ doping, improved luminescence lifetime	High red emission purity, water dispersibility, reduced quenching via NaYF_4_ shell	No direct therapeutic validation in biological models; limited to luminescence characterization	[181]
PTT	NaYF_4_:Yb,Ho,Ce@NaGdF_4_:Yb,Nd@NaGdF_4_ UCNP coated with polydopamine (UCNP@PDA)	Core-shell-shell NaYF_4_:Yb,Ho,Ce@NaGdF_4_:Yb,Nd@NaGdF_4_ structure with Ce^3+^ doping and PDA coating	Dual NIR excitation (980 nm and 808 nm); PDA absorbs both UCNP visible emission and external 808 nm light	Photothermal conversion leading to tumor cell ablation	In vitro cytotoxicity with rat bone marrow stromal cells (rBMSCs) and PTT on MG-63 osteosarcoma cells	Good biocompatibility (>90% viability in rBMSCs) and effective tumor cell killing (<15% viability in MG-63 under 808 nm irradiation)	Stable photothermal effect with PDA thickness variation; intense red emission for deep tissue imaging	Evaluation limited to in vitro models; no in vivo validation yet	[189]
Phototherapy (PDT/PTT)	UCNPs@mSiO_2_-MC540-FePc nanoplatform	NaErF_4_@NaYbF_4_@NaYF_4_ core-shell-shell UCNPs with mesoporous silica coating	1550 nm NIR-II excitation; Er^3+^ sensitization with dual red-green emission	Synergistic PDT (MC540, green emission) + PTT (FePc, red emission)	In vitro: HeLa cells; In vivo: nude mice tumor model	Significant tumor growth reduction by 8.34% with combined PDT/PTT	Deeper tissue penetration and reduced overheating compared to 980 nm excitation; high photothermal stability	Complexity of UCNP design and need for precise shell thickness/doping optimization; potential translational challenges	[185]
Light-triggered crosslinking for cancer therapy	LINC based on PFPA-functionalized lanthanide-doped UCNPs	NaGdF_4_:Yb,Tm@NaYF_4_:Yb@NaGdF_4_:Yb,Nd@NaGdF_4_ core-shell nanocomposite (Tm-CSSS) with PFPA surface modification	808 nm NIR excitation → UV UCL (200-400 nm) → PFPA activation for covalent crosslinking	Spatiotemporally controlled bioconjugation enabling cell-cell fusion, tumor targeting, and immune activation	In vitro: HEK293T, RAW264.7 macrophages, dendritic cells. In vivo: 4T1 tumor-bearing mice	10-fold tumor enrichment, efficient osteoclast generation, lymph node localized immune activation with spatial control	High tissue penetration, low cytotoxicity, precise spatiotemporal control of bioconjugation	Requires external NIR irradiation; complexity of multi-layer UCNP synthesis	[124]
Drug delivery and pharmacokinetic monitoring	UCNP-DNA nanocomplex (UCDC) signal processor	Core-shell NaYF_4_:Yb,Er,Tm@NaYF_4_ with DNA interfacial and epitaxial assembly	Activation MechanismNIR excitation (980 nm) with ratiometric imaging (UCLR/UCLNIR) and pH-responsive DNA transformation	Therapeutic ModalityReal-time monitoring of mitoxantrone (MTX) release	Breast cancer mouse xenograft model (MCF-7) and in vitro cell assays	Accurate, reliable localized drug-release monitoring with quantitative correlation between luminescence ratio and cumulative MTX release	Minimized influence of microenvironment fluctuations and dosage variation; robust ratiometric imaging	System complexity and potential DNA degradation in vivo before tumor targeting	[194]
Implantable delivery system	UCNP-coated organic photovoltaic (OPV) cells integrated with MEMS drug reservoirs	Core-shell NaYF_4_:Yb,Er@NaYF_4_:Nd,Yb UCNPs	NIR (808 nm) → UCNP emission (540 & 650 nm) → OPV photocurrent dissolves Au thin film	On-demand release of rhodamine B (model drug)	In vitro (PBS buffer, rhodamine B reservoirs)	Complete drug release within 20 min upon NIR irradiation	Implantable, flexible, wireless power source enabling spatiotemporal drug release	Currently limited to in vitro demonstration; requires optimization for in vivo implantation (penetration depth, safety, device location)	[195]
Theranostics (biodegradable nanocarrier)	Cellulose acetate (CA) encapsulated NaYF_4_:Yb,Er,Ce UCNPs loaded with doxorubicin	NaYF_4_:Yb^3+^,Er^3+^,Ce^3+^ UCNPs encapsulated in CA nanocapsules	NIR excitation (980 nm) → UCNP luminescence retained after encapsulation	pH-triggered release of doxorubicin + optical imaging	In vitro (L929 fibroblasts, MCF-7 breast cancer cells)	63% drug loading efficiency; pH-dependent release (47% at pH 3.6); reduced UCNP toxicity; effective inhibition of MCF-7 cells under acidic conditions	Biocompatible, stable, reduced toxicity, dual imaging + drug delivery	Only in vitro tested; luminescence intensity reduced ∼30% after encapsulation; requires in vivo validation	[196]
Cancer Therapy	Folate-receptor targeted Pluronic F127-TPGS micelles co-loaded with cisplatin (CDDP) and UCNPs, decorated with chitosan-folate (CHI-FA)	Rare-earth doped UCNPs (NaYF_4_:Yb,Er type, incorporated into micelles)	NIR excitation (980 nm) → UCNP luminescence for imaging; micelle-mediated cisplatin release	Chemotherapy (cisplatin) + imaging (theranostic)	In vitro (A549 lung cancer cells, hemocompatibility assays); In vivo (Wistar rats: pharmacokinetics, BAL fluid biochemistry, histopathology)	IC_50_ reduced ∼5.4-fold vs. free cisplatin; AUC increased ∼7.6-fold; prolonged half-life (up to 15.3 h); reduced hemolysis (<2%)	Targeted delivery via folate receptor; improved pharmacokinetics; reduced systemic toxicity; theranostic capability	UCNP encapsulation efficiency relatively low (∼32%); luminescence not deeply characterized; requires further clinical validation	[35]
PDT combined with nitric oxide (NO) gas therapy	Photoswitchable UCNPs@mSiO_2_ loaded with zinc phthalocyanine (ZnPc) and Roussin's black salt (RBS)	Core-multi-shell NaYF_4_@NaErF_4_:Tm@NaYF_4_@NaYF_4_:Yb,Ho,Nd@NaYF_4_@NaYF_4_:Yb,Tm with excitation-dependent trichromatic emission	Stepwise NIR excitation: 980 nm for NO release, 1550 nm for ROS generation, 808 nm for imaging guidance	Programmable cascaded phototherapy (NO release followed by PDT)	In vitro HeLa cells and in vivo 4T1 tumor-bearing nude mice	Sequential NO→ROS activation alleviated tumor hypoxia, enhanced PDT efficiency, and significantly inhibited tumor growth	Precise spatiotemporal control of multiple therapeutic processes with orthogonal NIR excitations	Complex fabrication requiring five shells; time-consuming and challenging synthesis	[197]
Oncotherapy via combined PDT and chemodynamic therapy	Er-doped NaYF_4_:Yb/Er UCNPs coated with DSPE-PEG liposomes, loaded with hypocrellin B, and in-situ grown MnO_2_ nanosheets	Phospholipid-coated NaYF_4_:Yb/Er UCNPs with DSPE-PEG_2000_ encapsulation, average diameter ∼28 nm	980 nm NIR excitation → UCNP green emission (545 nm) → HB J-aggregate absorption → ROS generation; MnO_2_ reacts with H_2_O_2_/GSH for O_2_ supply and Fenton-like OH production	Synergistic PDT (ROS generation) + chemodynamic therapy (Mn^2+^ mediated OH radicals)	In vitro: HeLa cells; In vivo: 4T1 tumour-bearing Balb/c mice	Combined PDT/CDT achieved ∼92.7% tumour growth inhibition in vivo; strong apoptosis induction in vitro	Overcomes hypoxia barrier via MnO_2_ oxygen self-supply; efficient energy transfer from UCNPs to HB; synergistic PDT/CDT efficacy	Requires intratumoral injection; potential thermal effects of 980 nm laser; limited systemic delivery data	[187]
Cancer nanomedicine	UCNP@Ale-PEG-Flamma® nanoparticles for pancreatic cancer	NaYF_4_:Yb,Er UCNPs coated with PEG-alendronate and conjugated with Flamma® dye	NIR excitation (980 nm) with LRETto Flamma®	Imaging and biodistribution tracking (potential drug delivery)	Orthotopic Panc02 murine pancreatic tumor model (C57BL/6 mice)	Intraperitoneal administration led to >6-fold higher tumor accumulation compared to intravenous	Enhanced tumor targeting and biodistribution with intraperitoneal route; improved imaging sensitivity	Mechanism of enhanced IP accumulation not fully understood; systemic circulation still observed	[198]
Cancer nanomedicine	Polypeptide copolymer-UCNP composite nanoparticles loaded with doxorubicin	Core-shell NaYF_4_:Tm^3+^, Yb^3+^/NaYF_4_ UCNPs embedded in polypeptide copolymer matrix	NIR (980 nm) converted to UV by UCNPs, triggering polymer disassembly	Controlled, pulsatile drug release (DOX delivery)	HeLa cells (in vitro cytotoxicity assays)	NIR irradiation reduced IC_50_ from 8.26 to 5.08 μg/mL (5 min) and 2.95 μg/mL (10 min)	Tunable, on-demand drug release with enhanced cytotoxicity	Demonstrated only in vitro; no in vivo validation yet	[199]
Chemotherapy drug delivery (solid cancer treatment)	UCNPs@cAMS-6@DOX&MS with lysozyme coating for controlled release	NaYF_4_:Yb,Er UCNPs (∼27.5 nm) encapsulated in mesoporous silica (AMS-6) core-shell, ∼200 nm spheres, ∼4 UCNPs per particle	NIR (980 nm) excitation triggering UCNP emission (UV/Vis) plus protein lysozyme coating for prolonged release	Chemotherapy agent DOX delivered via stimuli-responsive release	MCF-7 breast cancer cells in vitro	NIR irradiation accelerated DOX release (up to 92% in 3h); lysozyme coating slowed burst release and prolonged kinetics; effective cytotoxicity against cancer cells	Dual-function system enabling both promotion and inhibition of drug release; spatiotemporal control; reduced burst release	Evaluation limited to in vitro breast cancer cells; no in vivo validation yet	[200]
Targeted Drug Delivery /Theranostics (Cancer)	Alginate-PEG-folate functionalized UCNPs loaded with doxorubicin	NaLuGdF_4_:Yb,Er,Cr core UCNPs coated with nonionic alginate-PEG-folate polymers	Activation Mechanism NIR (980 nm) excitation with pH-responsive release via folate receptor-mediated endocytosis	Chemotherapy (doxorubicin delivery) combined with NIR imaging	KB nasopharyngeal carcinoma cells and HeLa cervical cancer cells	Enhanced DOX loading efficiency, controlled pH-responsive release, improved cytotoxicity against KB cells, and strong NIR imaging capability	High biocompatibility, improved drug loading, selective targeting via folate, and enhanced luminescence intensity	Evaluation limited to in vitro cell models; no in vivo validation provided	[201]
Theranostics (Imaging + Drug Delivery)	Orthogonal photoactivatable UCNP clusters (OP-UCNPs-C@mSiO_2_@azo-P)	Modularly assembled UCNP clusters (UCNPs A: NaYF_4_:60% Yb, 20% Gd, 2% Er@NaLuF_4_:25% Y; UCNPs B: NaYF_4_:30% Yb, 0.5% Tm@NaYF_4_:10% Yb@NaNdF_4_:10% Yb) coated with mesoporous silica and azobenzene caps	Dual NIR excitation: 980 nm → red emission (imaging); 808 nm → UV/blue emission (drug release via azobenzene cis-trans switching)	Paclitaxel delivery + fluorescence imaging	HeLa cells (2D cultures and 3D spheroids)	Orthogonal control achieved: imaging without drug release at 980 nm; drug release and cell killing at 808 nm	Independent programmable control of imaging and therapy; reduced off-target toxicity; modular and flexible synthesis	Larger cluster size reduces emission efficiency inside cluster; luminescence weaker compared to single UCNPs; synthesis modular but emission attenuation inside cluster	[202]

#### 5.3.2 Drug and gene delivery systems

In parallel with phototherapy, UCNPs have been extensively explored as carriers for controlled drug and gene delivery. These systems depend on the efficient generation of UV or visible photons via multiphoton ESA or ETU processes, which are then absorbed by photo-labile bonds or responsive matrices to trigger localized payload release. One approach involves light-triggered release mediated by photochemical cleavage. Wang et al. developed a dual-responsive system based on NaYF_4_:Yb,Er UCNPs encapsulated within a poly(o-nitrobenzyl) shell, enabling both NIR- and pH-controlled doxorubicin release [192]. Under combined irradiation and acidic conditions, the upconverted UV/visible light breaks the photosensitive polymer bonds, demonstrating how UCNP-mediated photochemical transformations can dictate release kinetics. Another strategy relies on structural gating mechanisms that respond to biological stimuli. Huang et al. designed a mesoporous silica-coated UCNP system with a lysozyme shell acting as a biological gatekeeper [200]. This architecture prevents premature drug release while enabling enzymatically triggered opening in tumor environments. More advanced delivery platforms incorporate real-time monitoring and programmable release, such as DNA-assembled UCNP nanocomplexes capable of ratiometric imaging of drug release in vivo [194]. Similarly, Hu et al. introduced a light-activated nanocrosslinker system that enables spatiotemporal control of biomolecule interactions through UCNP-generated UV emission [124]. These systems demonstrate the potential of UCNPs to function not only as delivery triggers but also as reporters of therapeutic processes. Additional approaches, including photoacid-assisted release systems [193], polymer-based nanocarriers [196], and orthogonally controlled UCNP clusters [202], further expand the design space of UCNP-mediated delivery and are summarized in Table 5. While these systems offer high levels of control, they also highlight a key limitation: increasing functional sophistication often leads to complex architectures that may be difficult to reproduce and scale.

#### 5.3.3 Therapeutic performance and design trade-offs

Across therapeutic applications (summarized in Table 5), UCNP systems exhibit a clear transition from single-function platforms toward highly integrated theranostic architectures. Phototherapy-based systems leverage emission engineering to achieve localized activation of therapeutic agents, while delivery systems exploit UCNP-mediated photochemical or environmental triggers to control release. The most advanced platforms combine these approaches, enabling simultaneous imaging, therapy, and monitoring. However, while these complex, multi-component systems demonstrate remarkable therapeutic outcomes in controlled in vitro and small-animal models, they suffer from severe photophysical and biological constraints that hinder clinical translation. The multi-photon nature of upconversion inherently results in low quantum yields. Consequently, massive localized laser irradiance is often required to generate sufficient photons to trigger drug release, or to produce enough ROS and heat for effective tumor ablation. These required power densities frequently exceed the maximum permissible exposure (MPE) limits for clinical human use, risking severe collateral tissue damage. Furthermore, from a structural perspective, these systems are highly over-engineered. Complex core-multi-shell architectures loaded with mesoporous silica, targeting peptides, and dual photosensitizers almost always push the hydrodynamic diameter well beyond the renal clearance threshold. As a result, the most therapeutically potent UCNPs are also the ones most likely to persist indefinitely in the liver and spleen. Future progress must pivot away from simply adding more functional components to a single particle. Instead, research must focus on maximizing the non-radiative energy transfer efficiency directly from the lanthanide lattice to the photosensitizer (e.g., via plasmonic coupling or minimized interfacial distances) to allow effective therapy at clinically safe irradiation limits.

### 5.4 Remote biological control and emerging smart systems

Beyond sensing, imaging, and therapy, UCNPs enable a distinct class of biomedical functionalities based on the remote and programmable control of biological processes. In these systems, UCNPs are not merely passive reporters or energy donors; they are active transducers that convert external NIR inputs into biologically meaningful localized optical signals capable of modulating cellular behavior, gene expression, or biochemical circuitry. This capability arises from their unique combination of wavelength-selective excitation, tunable energy transfer pathways, and long-lived excited states, which together provide a platform for orthogonal activation and precise spatiotemporal control.

Unlike therapeutic systems, where the goal is to induce localized chemical effects such as heat or ROS generation, remote control platforms require tight coupling between UCNP emission and biological response mechanisms. As a result, design priorities shift toward emission programmability, excitation selectivity, and compatibility with light-responsive biomolecules or engineered biological systems. These requirements have led to the emergence of two closely related directions: optogenetic and biohybrid control systems, and more advanced logic-gated or data-driven UCNP platforms.

#### 5.4.1 Optogenetic and biohybrid control systems

Optogenetic systems represent one of the most direct applications of UCNP-mediated remote control. Conventional optogenetics relies on visible light (typically blue or green), which suffers from severe tissue attenuation and requires invasive fiber-optic delivery. UCNPs circumvent this by converting deeply penetrating NIR light into localized visible emission right at the target site allowing for a minimally invasive activation process in deep tissue environments.

A representative example is the UCNP-based biohybrid system developed by Xu et al., where genetically engineered *E. coli* were coupled with orthogonally emissive UCNPs to enable light-controlled bacterial motility [203]. In this system, distinct UCNP architectures emit either blue or green light under different excitation wavelengths (980 nm and 808 nm). Mechanistically, this relies on the spatial isolation of distinct ETU networks within the nanocrystals, ensuring that the 980 nm and 808 nm excitation pathways remain completely orthogonal without cross-talk. This dual-wavelength control activates separate optogenetic pathways regulating chemotaxis proteins, enabling reversible switching between bacterial swimming and tumbling states [203].

**Fig. 11:**
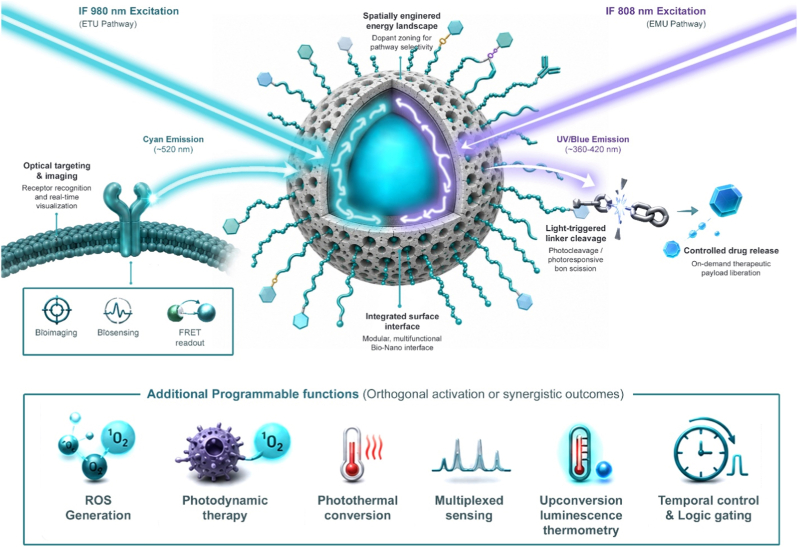
Orthogonal excitation and programmable optical logic in “smart” UCNP systems. Spatially engineered UCNP architectures execute distinct biological functions via wavelength-selective activation, utilizing 980 nm excitation (ETU pathway) for real-time bioimaging and 808 nm excitation (EMU pathway) for light-triggered drug release. This dual-input/dual-output capability transforms UCNPs from passive optical probes into dynamically programmable nanodevices capable of executing basic molecular logic.

This paradigm of wavelength-dependent modulation relies on spatially engineered UCNP architectures where distinct energy transfer networks are strictly isolated (Fig. 11). For example, 980 nm excitation can be routed through ETU pathways to yield visible emission (e.g., cyan/green) dedicated to real-time optical targeting, receptor recognition, and biosensing. Orthogonally, 808 nm excitation can trigger independent EMU pathways that generate localized high-energy UV or blue photons. These photons act as precise nanoscale triggers to actively cleave photo-responsive surface linkers, liberating therapeutic payloads on demand. This coexistence of multiple, non-interfering, excitation-dependent outputs allows researchers to seamlessly integrate multiplexed sensing and precise spatiotemporal drug delivery within a single unified nanoscale architecture.

Another direction involves UCNP-enabled neural and physiological modulation. Sun et al. demonstrated the wireless activation of dopamine neurons using UCNP-doped hydrogels implanted in the brain [204]. Here, efficient ETU pathways convert 980 nm excitation into a localized blue emission intensity sufficient to cross the optical activation threshold of channelrhodopsin-2, effectively modulating behavior in depression-model mice [204]. Similarly, Yang et al. developed a UCNP-cell hybrid system interfaced with graphene transistors, enabling infrared sensing through optogenetically induced ion flux [205]. These studies demonstrate that by finely tuning dopant concentrations to maximize the radiative output of specific transitions, UCNPs can function as reliable optical intermediaries between external IR stimuli and voltage-gated biological machinery. Despite these advances, optogenetic UCNP systems face several limitations. Efficient activation requires sufficient emission intensity under biologically safe excitation conditions, while maintaining compatibility with sensitive biological environments. In addition, the integration of UCNPs with living systems introduces challenges related to stability, immune response, and long-term functionality. These factors highlight the need for improved material design and system simplification to enable broader applicability.

#### 5.4.2 Programmable logic-gated and data-driven UCNP systems

Building on optogenetic control, recent developments have expanded UCNP functionality toward programmable and information-rich systems where signal generation or therapeutic action depends on computational processing or logic-gated inputs.

Logic-gated UCNP systems aim to improve specificity by requiring coordinated inputs before producing an output. Yan et al. demonstrated a UCNP-supported elemental selenium platform that integrates multiple therapeutic modalities within an AND logic framework, where activation depends on the simultaneous presence of specific microenvironmental triggers [206]. This approach drastically reduces off-target effects and enhances selectivity by ensuring that therapeutic responses occur only under defined conditions. Similarly, Chao et al. developed DNA-based logic circuits coupled with UCNP emission, enabling multistep molecular computation for accurate cancer cell imaging [207]. As illustrated in the functional summary of (Fig. 11), this orthogonal control effectively transforms the UCNP from a passive luminescent probe into a dynamically programmable logic gate (e.g., IF 980 nm THEN target/image; IF 808 nm THEN release). This architecture reduces off-target effects and enhances selectivity by ensuring that therapeutic responses occur only under defined, wavelength-specific conditions.

Machine learning introduces a complementary dimension to UCNP-based systems by enabling the interpretation and optimization of complex, multi-layered optical signals. Liao et al. demonstrated that deep learning algorithms can classify the optical fingerprints of individual UCNPs with high accuracy, leveraging their multidimensional ETU emission characteristics [208]. This capability is particularly important for multiplexed sensing and imaging, where conventional analysis methods may fail to resolve overlapping signals. Liu et al. further extended this approach by combining lifetime-engineered UCNPs with deep learning-assisted super-resolution imaging, achieving high decoding accuracy in multiplexed systems [183]. By actively orchestrating EMU across multiple shells, they generated discrete lifetime decay profiles that machine learning algorithms could easily decode, enabling massive multiplexing. In addition to signal decoding, machine learning has also been applied to UCNP design. Xia et al. employed Bayesian optimization to identify multishell UCNP architectures with enhanced ultraviolet and violet emission, demonstrating how data-driven approaches can accelerate the optimization of highly complex non-radiative energy transfer networks, and the discovery of materials with tailored optical properties [209]. These advances suggest that future UCNP systems may integrate both programmable biological responses and computational optimization, enabling adaptive and highly efficient nanoplatforms.

#### 5.4.3 Functional performance and system complexity in smart UCNP platforms

Across remote control and smart system applications, UCNPs demonstrate a remarkable evolution from simple optical converters to active components of intelligent bio-circuitry for multifunctional platforms applications capable of mediating complex biological and computational processes. Optogenetic systems highlight the ability of UCNPs to enable minimally invasive, deep-tissue activation of biological pathways, while logic-gated and data-driven platforms introduce higher levels of selectivity and information processing. However, a highly critical analysis reveals that these systems are currently far from clinical translation due to severe photophysical and architectural bottlenecks.

First, most optogenetic channels (like channelrhodopsin) require high-energy blue or UV light for activation. While UCNPs successfully generate this light locally, blue/UV photons have a tissue penetration depth limited to mere micrometers. Therefore, the UCNP must be perfectly localized directly on the target cell membrane; any spatial mismatch renders the upconverted emission useless due to immediate absorption by surrounding biomolecules. Secondly, highly programmable platforms (such as those utilizing EMU lifetime-decoding or logic-gated delivery) require exceedingly complex core-multi-shell structures. The addition of each subsequent epitaxial shell inevitably introduces interfacial defects that act as non-radiative trapping centers, notoriously driving down the overall quantum yield of the particle. Consequently, these “smart” systems currently require intense, non-physiological laser power to function in vivo. Future progress must prioritize simplifying these architectures to restore quantum efficiency, and shifting optogenetic targets toward red-activatable channels to better align with the most efficient lanthanide emission pathways.

## 6 Challenges and future directions

The preceding sections have highlighted that, despite significant progress across sensing, imaging, therapy, and emerging smart systems, UCNP performance remains constrained by recurring limitations related to optical efficiency, surface interactions, system complexity, and translational feasibility. In particular, challenges such as insufficient brightness under biologically safe excitation, dependence on 980 nm irradiation, instability in physiological environments, limited clearance, and variability in synthesis protocols have been consistently observed across application domains [8,[210], [211], [212]]. While these issues manifest differently depending on the specific application, they ultimately converge into a set of broader, interconnected barriers that currently limit the clinical translation of UCNP-based technologies. Addressing these barriers requires a shift from application-specific optimization toward a more integrated and standardized framework for material design and implementation.

### 6.1 Optical and excitation constraints

Across all biomedical applications, the most fundamental limitation of UCNPs lies in their low luminescence efficiency under biologically acceptable excitation conditions. The widely used 980 nm excitation pathway, although effective for populating sensitizer states, introduces localized heating due to water absorption, thereby constraining safe operating power densities. Transitioning to 808 nm excitation through Nd^3+^ sensitization offers a partial solution by reducing photothermal effects, but often at the cost of reduced upconversion efficiency unless energy migration pathways and shell architectures are precisely engineered. This trade-off reflects a deeper physical limitation: upconversion is an inherently nonlinear process that requires sufficient photon flux to sustain efficient emission. As excitation power decreases to biologically safe levels, emission intensity declines disproportionately, limiting performance in imaging, sensing, and therapeutic activation. Although strategies such as multishell engineering, energy migration-mediated upconversion, and co-sensitization have improved efficiency, they frequently introduce additional complexity and reduce reproducibility. Moving forward, improving brightness at low irradiance is not simply an incremental optimization challenge, but a central requirement that will determine the viability of UCNPs in real biomedical environments.

### 6.2 Bio-nano interface and in vivo behavior

The interaction between UCNPs and biological environments remains one of the most significant barriers to reliable performance. Surface coatings such as PEG, silica, dextran, and zwitterionic layers improve colloidal stability and reduce nonspecific interactions, yet they do not fully prevent protein corona formation, aggregation, or immune recognition under physiological conditions. These processes directly influence biodistribution, targeting efficiency, and signal reproducibility, often leading to discrepancies between in vitro performance and in vivo outcomes. Moreover, the biological identity of UCNPs evolves dynamically after administration. Adsorbed biomolecules, ionic exchange processes, and local environmental conditions can alter surface properties and optical behavior in ways that are not fully understood. Establishing predictive relationships between surface chemistry, corona composition, and pharmacokinetics is therefore essential. Emerging strategies such as biomimetic coatings and cell membrane cloaking offer promising routes to improve circulation time and immune evasion, but their reproducibility and scalability must be demonstrated before they can be widely adopted [92,212].

### 6.3 Energy efficiency and physical limitations

Beyond excitation wavelength considerations, UCNPs face intrinsic energy efficiency constraints that affect all application domains. The requirement for sequential photon absorption leads to low quantum yields, particularly under low-power excitation, limiting both emission brightness and energy transfer efficiency to external agents such as photosensitizers or molecular probes. This bottleneck is especially critical in therapeutic and optogenetic systems, where insufficient emission can compromise biological activation. At the same time, attempts to improve efficiency through increased dopant concentration, complex shell architectures, or plasmonic coupling can introduce new challenges, including concentration quenching, spectral cross-talk, and structural variability. As a result, the field faces a fundamental balance between enhancing energy transfer efficiency and maintaining stable, reproducible material architectures. Addressing this limitation will require not only improved material design, but also a more quantitative understanding of how energy transfer processes operate under realistic biological conditions.

### 6.4 Complexity, reproducibility, and scalability

A defining trend in recent UCNP research is the increasing complexity of nanoparticle architectures. Multishell structures, multi-dopant systems, and multifunctional hybrid platforms have enabled remarkable advances in performance, yet often at the cost of reproducibility and scalability. Small variations in synthesis conditions can lead to significant differences in dopant distribution, shell thickness, and optical output, making it difficult to reproduce results across laboratories or scale production for practical applications. This trend raises a broader question regarding the balance between functionality and practicality. While highly engineered UCNP systems demonstrate impressive capabilities, their complexity may limit their translational potential. In contrast, simpler architectures, although potentially less versatile, may offer greater reliability and scalability. Establishing standardized protocols for synthesis, characterization, and reporting, including parameters such as quantum yield under defined excitation conditions, hydrodynamic size, and serum stability, is essential for improving comparability and accelerating progress toward application-driven design [8,212].

### 6.5 Translation barriers and realistic application pathways

Beyond material-level challenges, the translation of UCNP technologies is constrained by practical considerations related to instrumentation, regulatory frameworks, and clinical relevance. Many current systems rely on laboratory-scale excitation sources and detection setups that are not compatible with clinical or POC environments. Furthermore, achieving effective deep-tissue excitation often requires laser power densities that approach or exceed MPE limits for human tissue. Bridging this gap requires the co-design of UCNPs with compact, accessible hardware, such as diode lasers, portable detectors, and integrated optical systems, to ensure that optical performance achieved under controlled conditions can be realistically translated into clinical settings safely [30]. From a regulatory standpoint, UCNP-based systems face additional complexity due to the absence of well-defined approval pathways for inorganic luminescent nanomaterials. Regulatory agencies such as the U.S. Food and Drug Administration (FDA) and the European Medicines Agency (EMA) evaluate nanomaterials within existing frameworks for drugs, medical devices, or combination products. In this context, many UCNP platforms, particularly those integrating imaging, targeting, and therapeutic functionalities, fall into the category of combination products. This creates significant challenges in defining a primary mode of action, establishing appropriate preclinical models, and designing standardized evaluation protocols. Furthermore, compliance with established guidelines, such as ISO 10993 for biocompatibility and ICH recommendations for toxicological assessment, remains difficult due to UCNPs’ complex composition, multicomponent architectures, and dynamic surface properties.

A critical bottleneck in this regard is the lack of standardized and reproducible data on long-term safety and biological fate as discussed in previous sections. While many studies report low acute cytotoxicity, regulatory approval requires a comprehensive evaluation of biodistribution, degradation, and clearance pathways. A major paradox in UCNP design is the size-clearance trade-off: while nanoparticles typically must be under 5.5 nm for efficient renal clearance [213], scaling UCNPs down to this size severely quenches their quantum yield. Consequently, most high-performing UCNPs are larger and exhibit persistence in reticuloendothelial organs (such as the liver and spleen) [214]. The limited understanding of chronic exposure to these accumulated lanthanide-containing inorganic cores continues to hinder risk assessment and regulatory acceptance.

From a regulatory perspective, consistency is not limited to laboratory-scale synthesis, but requires the establishment of standardized, Good Manufacturing Practice (GMP)-compliant production processes that ensure batch-to-batch uniformity in physicochemical and functional properties. This includes strict control over parameters such as particle size distribution, surface chemistry, dopant composition, and optical performance, all of which must remain within defined specifications across large-scale production. In addition to safety considerations, scalability and manufacturing reproducibility represent major barriers. Highly engineered UCNP architectures, including multishell systems, rely on tightly controlled synthetic conditions (e.g., high-temperature thermal decomposition) that are difficult to reproduce at an industrial scale. While emerging techniques like microfluidic or continuous-flow synthesis show promise, current regulatory frameworks require robust manufacturing processes that guarantee consistent physicochemical properties across production batches. The lack of harmonized reporting standards for key parameters, such as quantum yield under biologically relevant excitation, hydrodynamic size distribution, and surface chemistry, further limits comparability between studies and slows translational progress.

These challenges become particularly evident when compared to clinically approved nanomaterials, such as liposomal drug carriers or iron oxide nanoparticles, which achieved regulatory approval through relatively simple, well-characterized compositions and clearly defined mechanisms of action. In contrast, UCNP systems often combine multiple functionalities within a single platform, increasing both their therapeutic potential and their regulatory burden. This suggests that simplified and application-specific UCNP designs offer a more realistic pathway toward clinical adoption than highly multifunctional constructs. As a result, near-term translation is highly likely to occur in localized or *ex vivo* applications. Formats such as time-gated lateral flow assays, targeted intraoperative imaging, and endoscopic diagnostics minimize systemic exposure and make regulatory requirements far more manageable. Aligning UCNP design with these realistic use cases, while progressively addressing safety, standardization, and manufacturing challenges, will be essential for bridging the gap between laboratory innovation and clinical implementation.

### 6.6 Future directions: toward application-driven UCNP systems

The future development of UCNPs will depend less on the discovery of new material compositions and more on the integration of existing knowledge into coherent, application-driven design frameworks. A key transition is expected from material-centered optimization toward performance targets defined by specific biomedical applications. This shift will require establishing clear benchmarks for brightness, sensitivity, and therapeutic efficacy under biologically relevant conditions, enabling more meaningful comparison and rational design.

Simplification of UCNP architectures is likely to become an important design principle. Rather than pursuing increasingly complex multifunctional systems, future efforts may focus on modular or task-specific platforms that balance performance with reproducibility and scalability. In parallel, the integration of UCNPs with device-level innovations, including portable excitation sources and real-time feedback systems, will play a crucial role in enabling practical deployment.

Data-driven approaches are also expected to accelerate progress in UCNP design. Machine learning and optimization algorithms can assist in identifying optimal dopant combinations, shell configurations, and excitation strategies, reducing reliance on empirical trial-and-error methods. Furthermore, the convergence of UCNP technology with synthetic biology and responsive materials may enable the development of adaptive systems capable of dynamically responding to biological signals. Ultimately, the successful translation of UCNPs into biomedical applications will require a coordinated effort that combines advances in materials science, surface engineering, device integration, and standardization. By aligning design strategies with realistic clinical requirements and emphasizing reproducibility and efficiency, UCNPs can evolve from promising nanoscale tools into robust and reliable platforms for next-generation biomedical technologies.

## 7 Conclusion

Over the past two decades, UCNPs have transitioned from fundamental photophysical systems to highly adaptable platforms for biomedical applications. Their ability to convert NIR excitation into higher-energy emission enables unique capabilities in deep-tissue imaging, low-background detection, and multiplexed analysis. When combined with advances in structural design and surface engineering, UCNPs have evolved into multifunctional systems that support sensing, imaging, therapy, and emerging forms of remote biological control. A key outcome of this progress is the recognition that UCNP performance is not governed by a single parameter, but by the interplay between photophysical mechanisms, material architecture, interfacial chemistry, and biological environment. Throughout this review, we have emphasized that effective UCNP design requires a coordinated approach that links these elements, rather than optimizing them in isolation. Rather than serving as universal solutions, UCNPs are most impactful when applied in contexts where their distinct properties provide clear advantages over conventional probes. In such settings, their various features enable capabilities that are difficult to achieve with other nanomaterial systems. Continued progress in this field will depend on maintaining this integrated design perspective, where material development, functional performance, and application requirements are considered simultaneously. With this approach, UCNPs are well positioned to move beyond proof-of-concept demonstrations toward more reliable and application-driven biomedical technologies.

## CRediT authorship contribution statement

**Faezeh Ghorbanizamani:** Data curation, Formal analysis, Investigation, Writing – original draft. **Hichem Moulahoum:** Data curation, Formal analysis, Investigation, Software, Writing – original draft, Writing – review & editing. **David John Dmonte:** Data curation, Formal analysis, Resources, Writing – review & editing. **Kalim Deshmukh:** Conceptualization, Funding acquisition, Project administration, Resources, Validation, Visualization, Writing – review & editing.

## Declaration of competing interest

The authors declare that they have no known competing financial interests or personal relationships that could have appeared to influence the work reported in this paper.

## Data Availability

The preprint copy of this article is available at: https://doi.org/10.5281/zenodo.19469510 and the dataset is available at: https://doi.org/10.5281/zenodo.19470014.
